# Supplementary material for: Say their names: Resurgence in the collective attention toward Black victims of fatal police violence following the death of George Floyd
Source: PLoS One. 2023 Jan 11;18(1):e0279225. doi: 10.1371/journal.pone.0279225 (PMC9833594; doi:10.1371/journal.pone.0279225)
Supplement: S1 Appendix — (PDF) [file pone.0279225.s001.pdf]

S1 Data Preprocessing

We take all fatalities of Black people in the Fatal Encounters database [53] from January 1, 2009 onward. There are 5,546 names that match these criteria. After removing deaths by suicide and vehicular and pursuit deaths, we are left with 3,897 cases. We parse the name of each Black victim into a 2-gram (two words) by removing periods, quotation marks, commas, and parentheses throughout the name, removing "Jr.," "Sr.," and Roman numerals from the end of each name, and taking the first and last word in each name. This is done to reflect common name conventions, although this method misses people who go by nicknames or middle names. In instances of duplicate names, where two or more individuals who suffered fatal police violence share a name, we attribute all the mentions of the name to the earlier of the two incidents. In total, there are 54 duplicate names. Another 95 names are excluded because they received measurable attention in the 10 days prior to their death, i.e. they were among the top million 2-grams in the 10 days prior. (One of those names, Walter Scott, was added back in manually.) We manually remove 12 cases of ambiguous names that are not captured by the previous step. Finally, 14 cases with unknown names are excluded, leaving 3,722 cases from the Fatal Encounters database. We list all of the names that were excluded in Tables S1-S4.

We expand the dataset with 15 additional names that are not included in the database and their dates of death to reflect key events relating to police brutality and anti-Black violence (giving a final count of 3,737 names). Because these names are chosen manually, they exhibit a relatively high level of attention. See Table S5 for a full list of manually selected names in the database.

S2 Mathematical Definitions

If $f_{\tau,t}$ is the raw frequency of a name τ on a given day t, then its relative frequency $p_{\tau,t}$ is

$$p_{\tau,t} = \frac{f_{\tau,t}}{\sum_{\tau' \in \mathcal{W}_t} f_{\tau',t}}, \tag{1}$$

where $\mathcal{W}_t$ is the collection of all 2-grams used on that day. If a name does not receive measurable attention, then we say that $p_{\tau,t}$ is 0.

We use the peak attention to construct the *normalized attention*

$$\widehat{p_{\tau,t}} = \frac{p_{\tau,t}}{\max_{t'} p_{\tau,t'}}. \tag{2}$$

a value between 0 and 1, where 0 indicates a name was not used within the top million 2-grams for a day, and 1 indicates the day that the most relative attention was given to a name since the date of death.

We measure the amplification of each name by distinguishing between how often they were used in originally authored tweets (OT) and retweets (RT), where we include the novel part of quote retweets among originally authored tweets. We operationalize amplification as the ratio between the two frequencies for a given name, namely $R_{\tau,t}$. This ratio is 1 when a name is used equally often in originally authored tweets and retweets. If $R_{\tau,t}$ is greater than 1, then the name is amplified via retweets more often than it is written itself, and vice versa if $R_{\tau,t}$ is less than 1.

$$R_{\tau,t} = \frac{f_{\tau,t}^{(\mathrm{RT})}}{f_{\tau,t}^{(\mathrm{OT})}}. \tag{3}$$

We define the *relative social amplification* [55, 60] as

$$R_{\tau,t}^{\mathrm{rel}} = \frac{R_{\tau,t}}{\sum_{w' \in \mathcal{W}_t} f_{w',t}^{(\mathrm{RT})} / \sum_{w' \in \mathcal{W}_t} f_{w',t}^{(\mathrm{OT})}}. \tag{4}$$

When retweets increase generally, the sum of all language used in retweets will increase, and so the denominator will increase as a whole. This adjusts for how $R_{\tau,t}$ may itself increase because of such increases in retweets. So, unlike $R_{\tau,t}$, the relative social amplification $R_{\tau,t}^{\mathrm{rel}}$ is comparable over wide time frames on Twitter, allowing us to compare the amplification of names to themselves and one another over time.

S3 Word Contributions to Expressed Happiness

In the main text, we find that the happiness expressed on Twitter following George Floyd's death reached historically low levels. This is based off a dictionary-based sentiment analysis approach. We use the labMT sentiment dictionary [57], which assigns scores to words based on how much "happiness" is associated with them. We use an updated version of the dictionary that also includes scores for words related to the pandemic and COVID-19. To calculate the average expressed happiness, we first count how often all the words in the labMT dictionary are used in tweets for a day or period of interest. These counts are treated as a single, large bag of words. We then use the labMT scores to calculate the weighted average, which is the average happiness.

The average happiness is just one number. We can better understand why it dropped in the wake of George Floyd's death by looking at how particular words contributed to that drop. To do so, we distinguish between different ways that a word can contribute. A word can contribute to the decrease in happiness if it is a *relatively negative* word ($-$) that is used *more frequently* ($\uparrow$). It can also contribute to the decrease if it is a *relatively positive* word ($+$) that is used *less* ($\downarrow$). On the other hand, a word can be counter to the decrease in happiness if it is a *relatively positive* word ($+$) that was used more ($\uparrow$), or a *relatively negative* word ($-$) that was used less ($\downarrow$). We treat the week prior to George Floyd's death as a reference period. We say that a word is *relatively* positive or negative if its labMT score is higher or lower than the average happiness of the reference period. We visualize the word contributions as word shift graphs [57,58], vertical bar charts showing the magnitude of how much each word contributes to the decrease in happiness following George Floyd's death. Deep yellow bars indicate relatively positive words that were used more ($+\uparrow$), deep blue bars indicate negative words that were used more ($-\uparrow$), light yellow bars indicate positive words that were used less ($+\downarrow$), and light blue bars indicate negative words that were used less ($-\downarrow$). The relative magnitude of each type of word contribution is shown at the top of the word shift graphs.

Figure S1 shows the word shift graph for May 31, 2020, the "saddest" day (day with the least happiness expressed) ever recorded by the Hedonometer, about a week after George Floyd's death. By the top of Fig. S1, we can see that increases in the frequency of negative words form the largest contribution to the change in happiness on this day. Increases in positive words and decreases in negative words make up the smallest contributions, and they are about equal. We observe that words such as "terrorist," "protest," "violence," and "racist" make large contributions as negative words that were used more. They relate to the protests following George Floyd's death and general conversation surrounding the topic of racism. Relatively happy words relating to "peace" contribute to shifting the happiness change in the other direction. Many coronavirus-related terms, which are relatively negative, were used less frequently, having been displaced by the protest- and racism-related terms.

We also show word shift graphs for three other specific days: the day following George Floyd's death (May 26th, 2020, see Figure S2), several days following his death (May 29th, see Figure S3), and the final day of the spike period (June 7th, see Figure S4). Immediately following his death, we see increased use of negative words relating to that death, like "murder," "killed," and "died" as well as terms related to the specific incident like "fraud" and "down." Note the words "dog," "dogs," and "park": on the day of George Floyd's death, May 25th, there was also a video-recorded incident in which a white woman walking a dog called the police on a Black man who was simply birdwatching in Central Park in New York City. By May 29th, those negative words have shifted to more protest-related terms, like "protesters," "riot," "thug," and "tear." Note, on both May 26th and the 29th, pandemic related words like "coronavirus," "lockdown," and "quarantine" were used less. Also by the 29th, we see some words being used more, like "justice" and "white," suggesting shifts to broader discussions of racial

justice. These words still see increase usage by the end of the spike period, relative to the week prior to the spike, and other words like “slave” and “slavery” were used more.
