# Supplementary figures and images for: Say their names: Resurgence in the collective attention toward Black victims of fatal police violence following the death of George Floyd
Source: PLoS One. 2023 Jan 11;18(1):e0279225. doi: 10.1371/journal.pone.0279225 (PMC9833594; doi:10.1371/journal.pone.0279225)

2020-05-19 to 2020-05-25:  $\Phi_{avg} = 5.95$   
2020-05-31:  $\Phi_{avg} = 5.63$

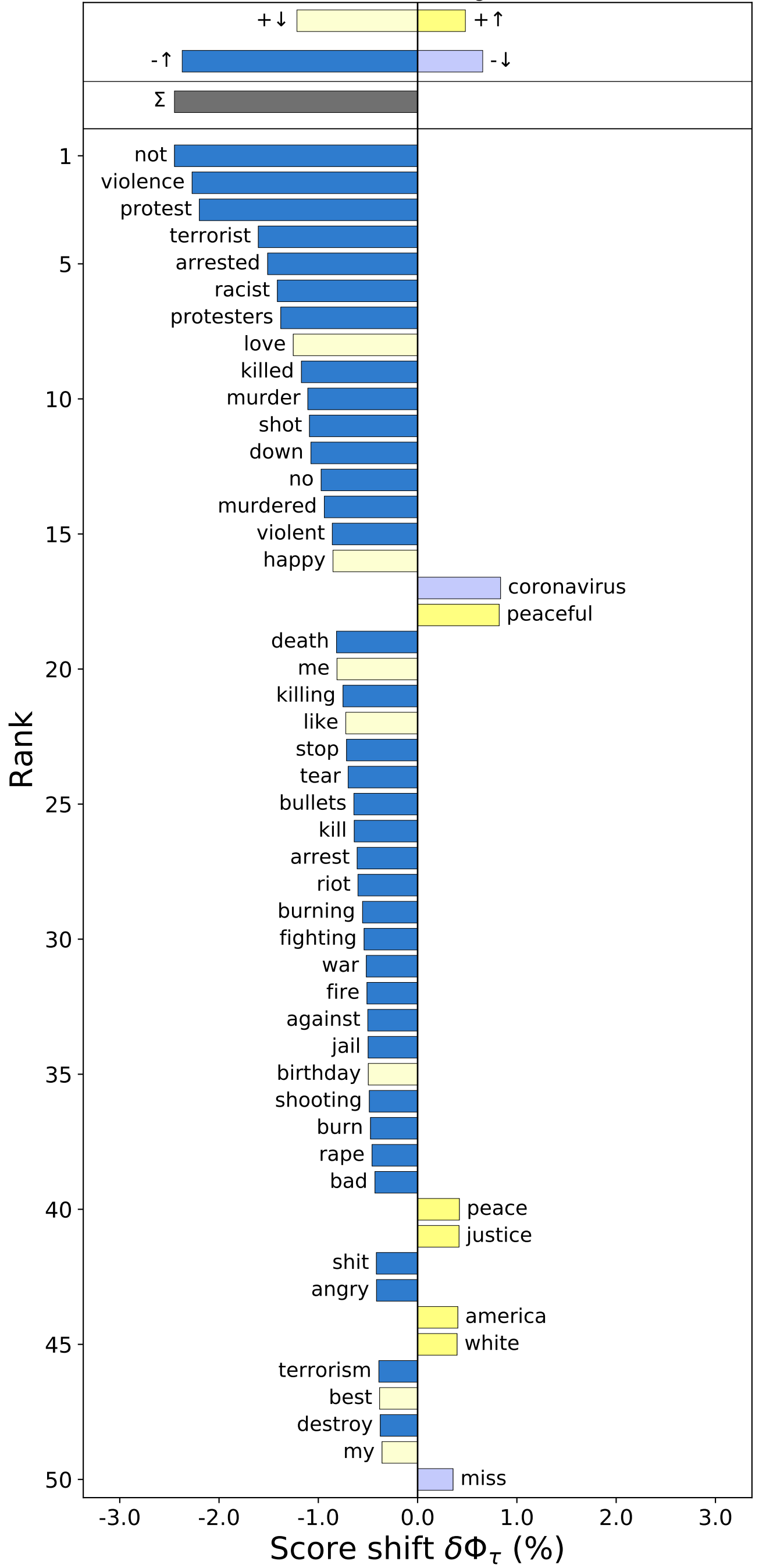

Supplement: S1 Fig — The week prior to George Floyd’s death is used as a reference period. (PDF) [file pone.0279225.s002.pdf]

2020-05-19 to 2020-05-25:  $\Phi_{avg} = 5.95$   
2020-05-26:  $\Phi_{avg} = 5.87$

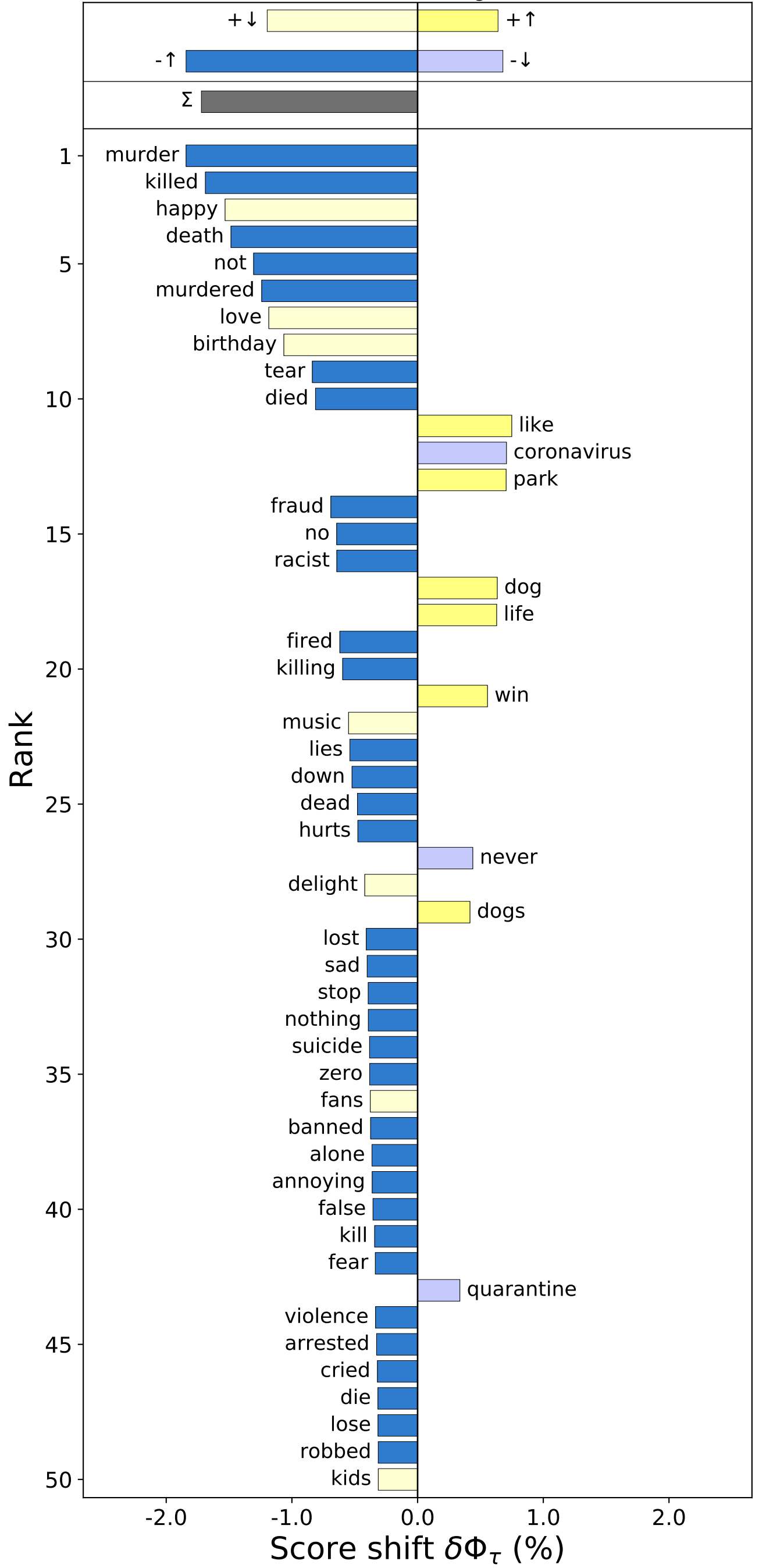

Supplement: S2 Fig — The week prior to George Floyd’s death is used as a reference period. (PDF) [file pone.0279225.s003.pdf]

2020-05-19 to 2020-05-25:  $\Phi_{avg} = 5.95$   
2020-05-29:  $\Phi_{avg} = 5.66$

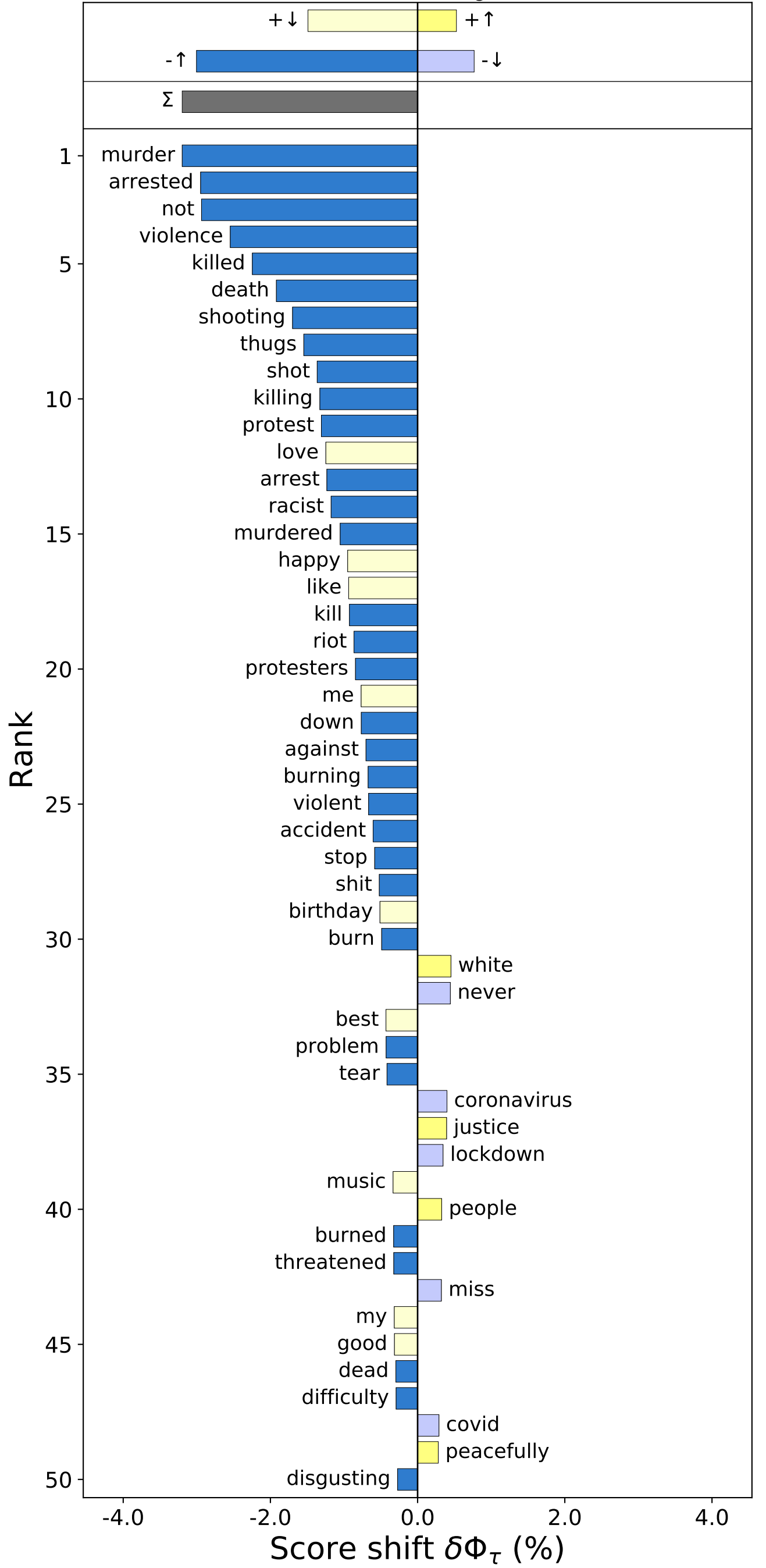

Supplement: S3 Fig — The week prior to George Floyd’s death is used as a reference period. (PDF) [file pone.0279225.s004.pdf]

2020-05-19 to 2020-05-25:  $\Phi_{avg} = 5.95$   
2020-06-07:  $\Phi_{avg} = 5.80$

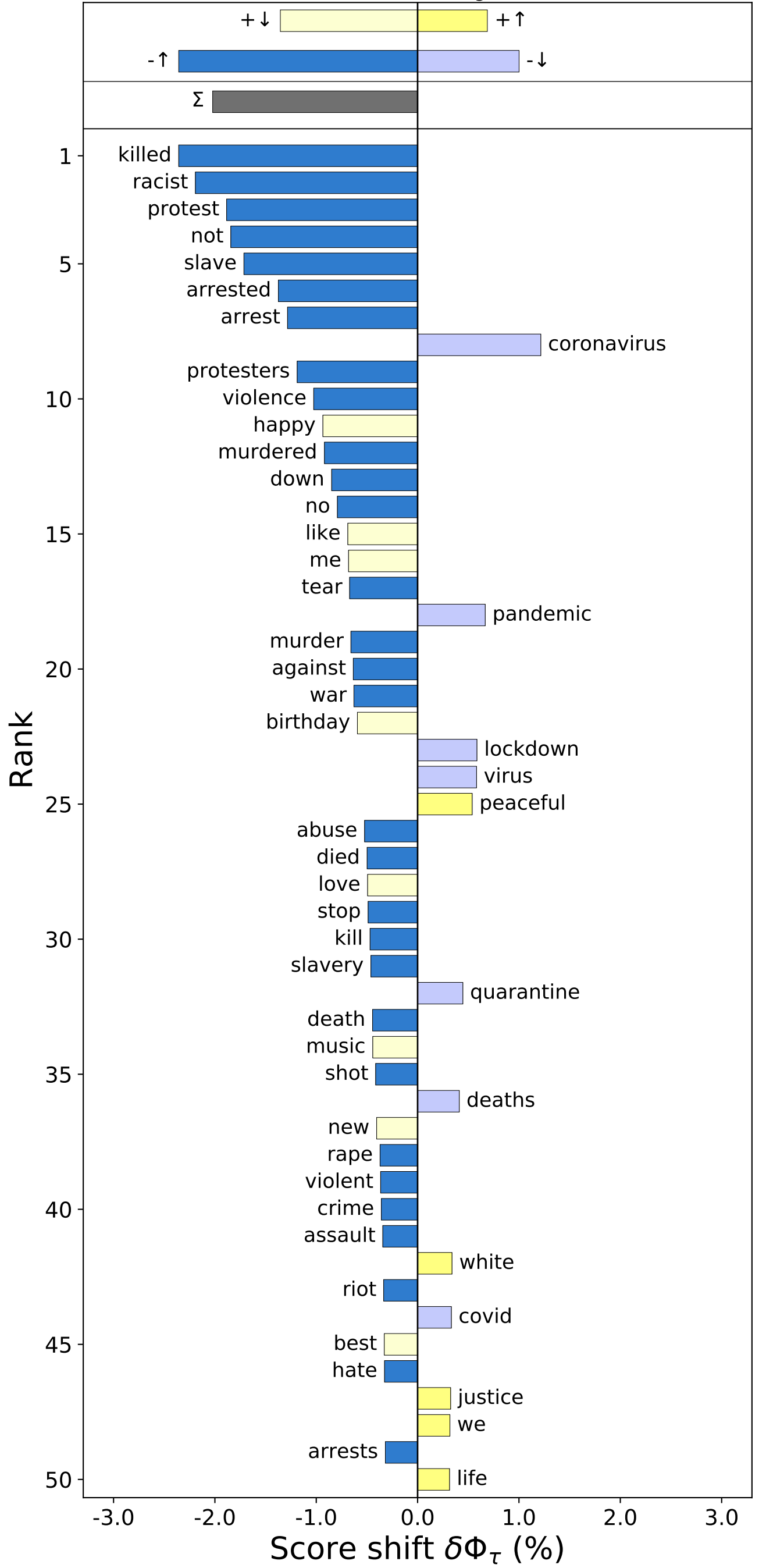

Supplement: S4 Fig — The week prior to George Floyd’s death is used as a reference period. (PDF) [file pone.0279225.s005.pdf]

Normalized frequency

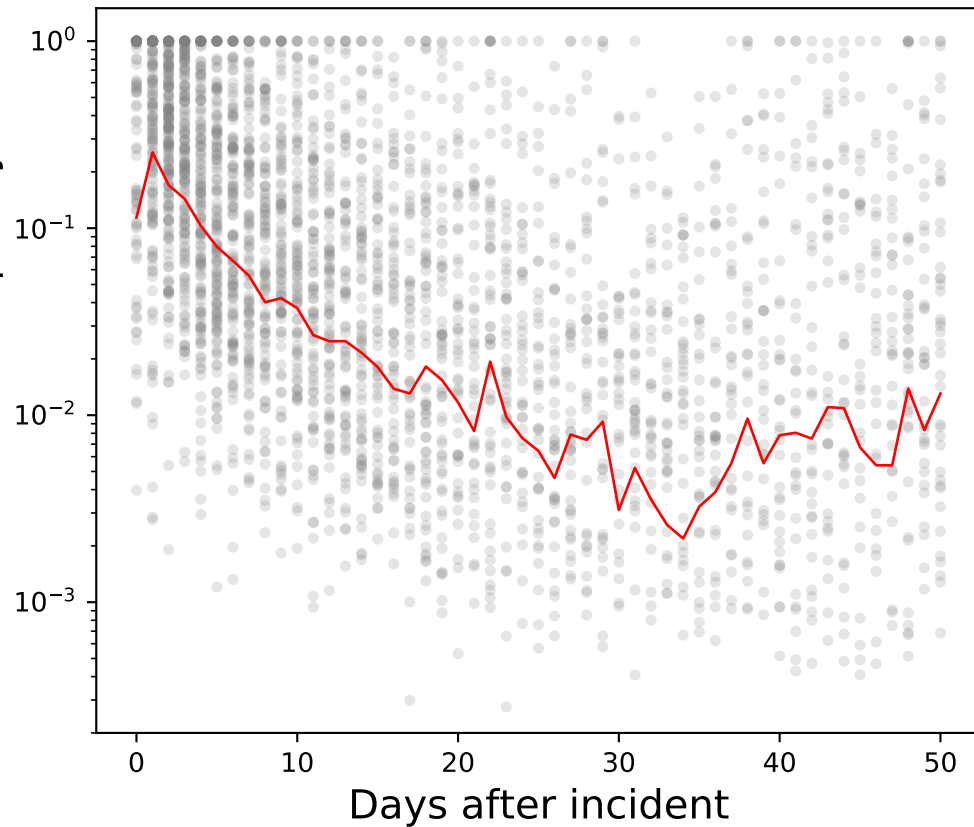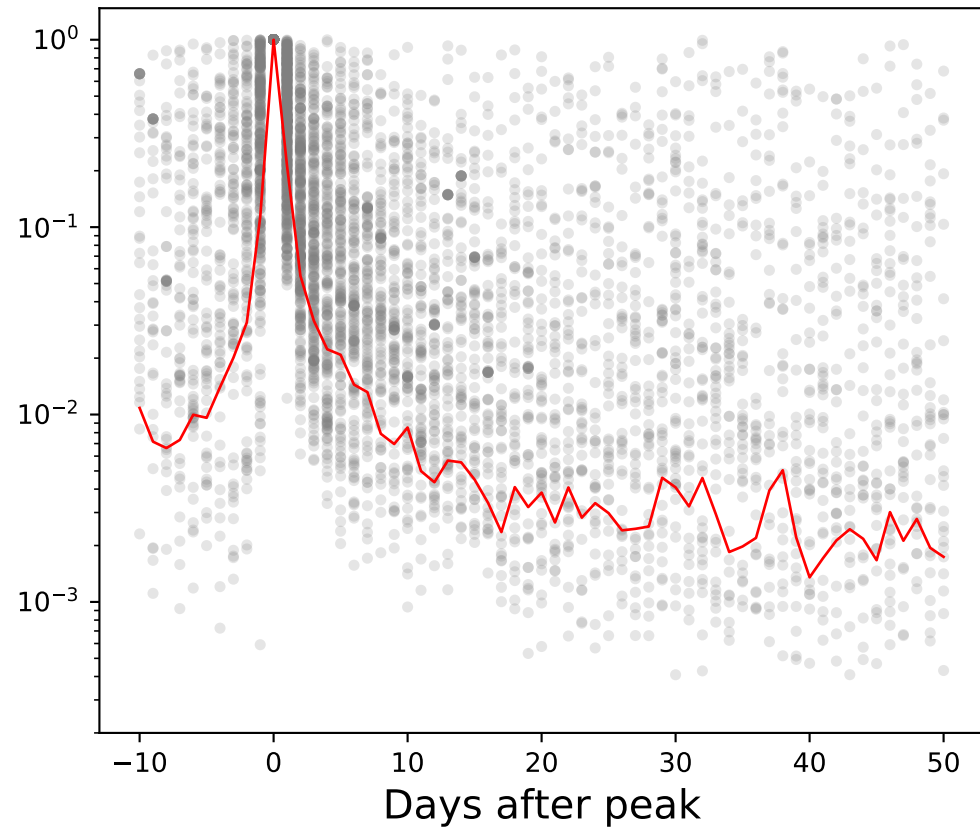

Supplement: S5 Fig — Scatter plots of normalized attention pτ,t^ for all Black victims in the combined database anchored to the first 50 days after the date of death (left) and peak relative frequency (right). We exclude names that did not receive measurable attention during the specified period, i.e. did not appear in the top million 2-grams. The red line plot shows the mean of the included values on each day. Because of the log-scaled y-axis, points with a normalized frequency of 0 are not visible but are included in the mean. (PDF) [file pone.0279225.s006.pdf]

Normalized frequency

Male

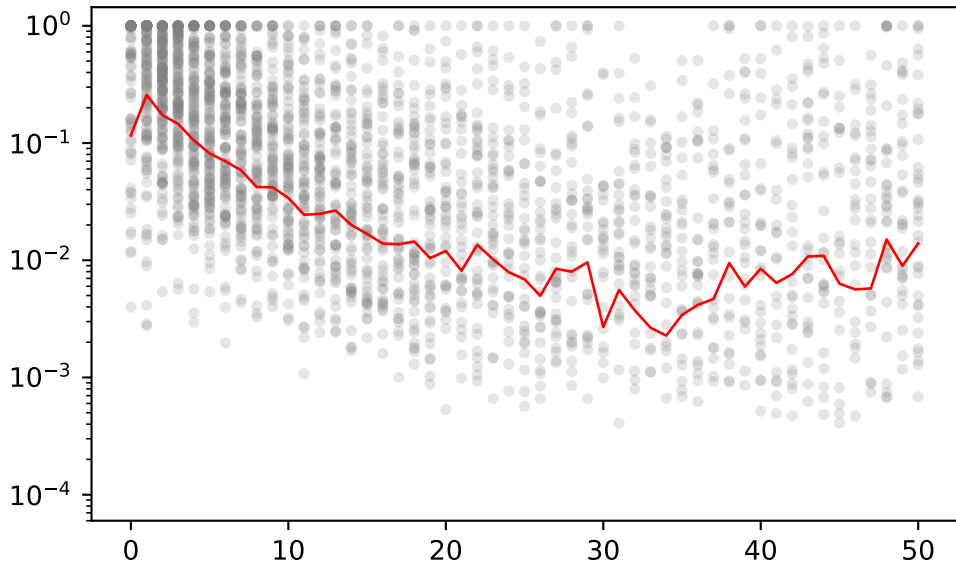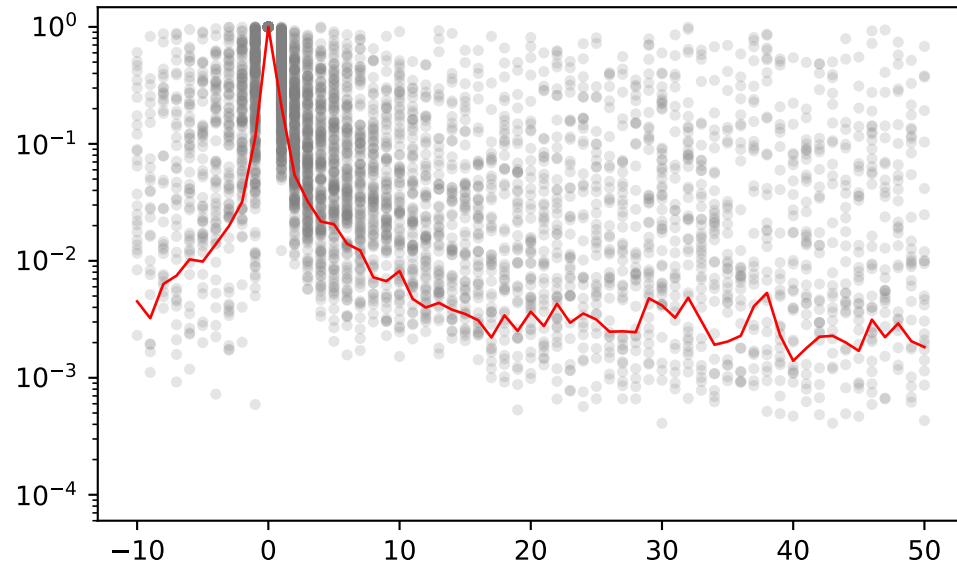

Female

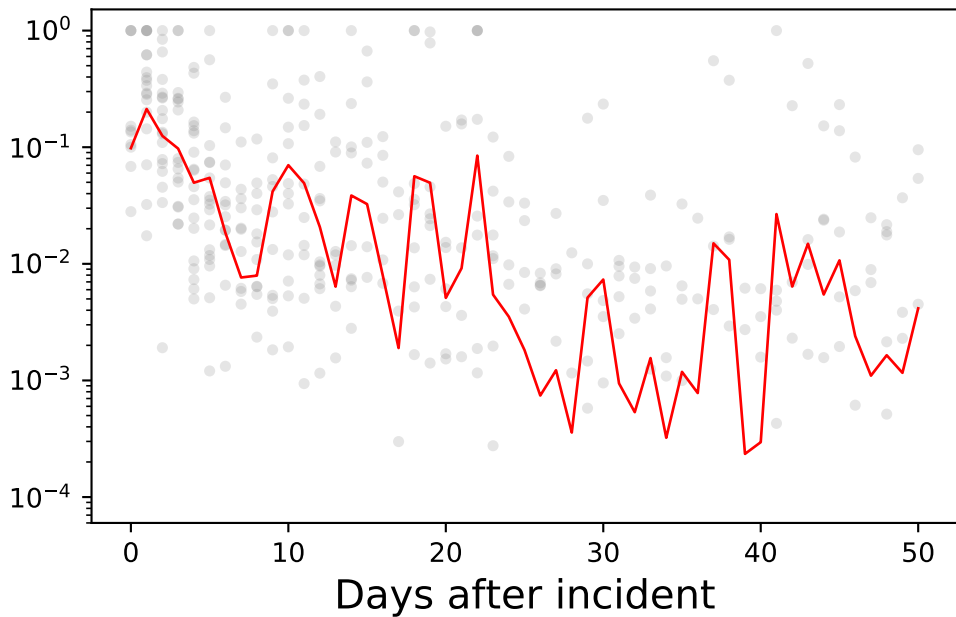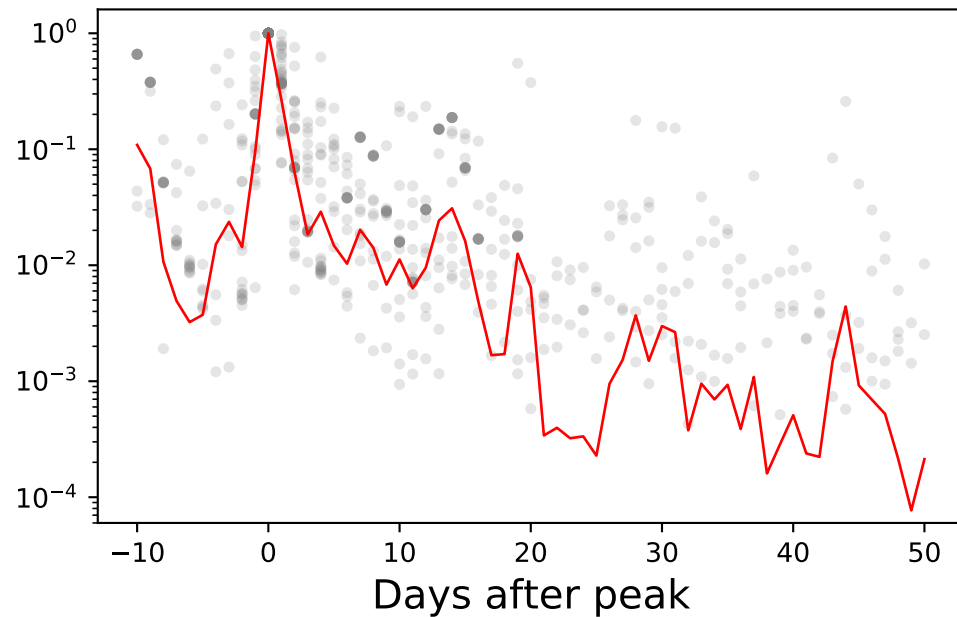

Supplement: S6 Fig — See S5 Fig for details. (PDF) [file pone.0279225.s007.pdf]

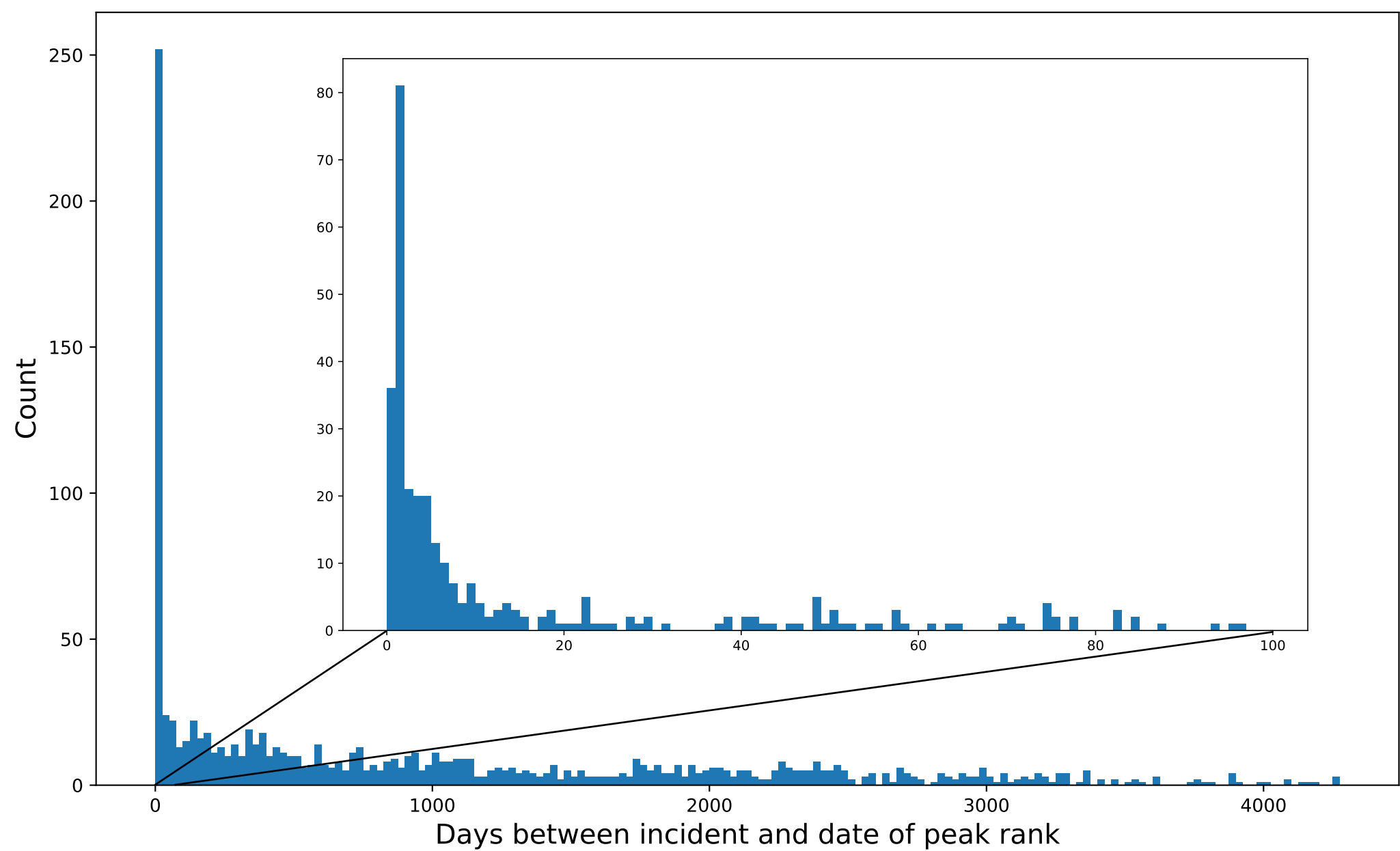

Supplement: S7 Fig — The main figure uses 25-day bins, while the inset uses 1-day bins from 0 to 100 days between incident and peak rank. Short delays are more common than long delays, with 0 and 1 days between the incident and the peak rank of a name being the most common. This provides evidence that most names in our analyses are properly disambiguated. (PDF) [file pone.0279225.s008.pdf]

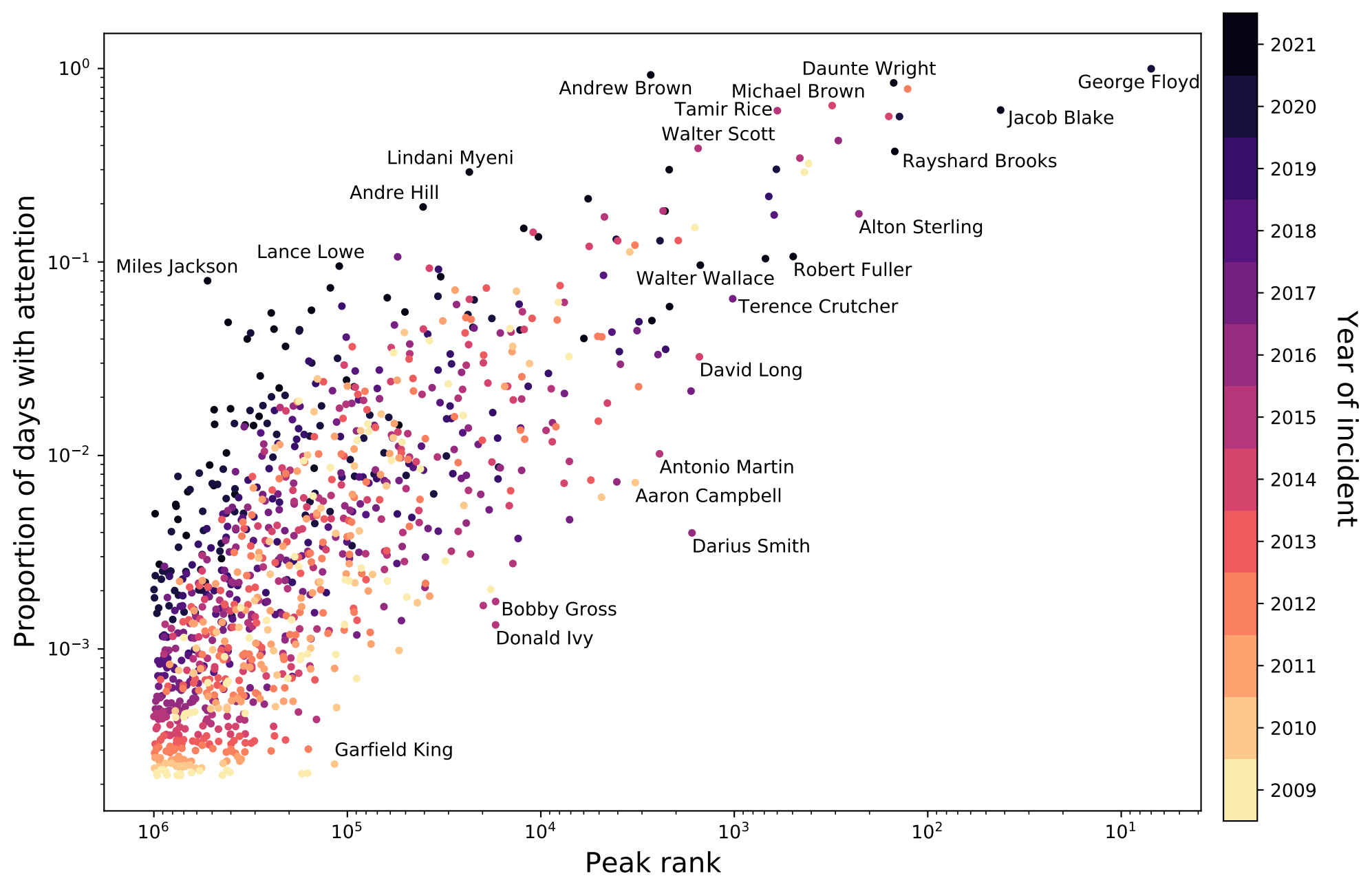

Supplement: S8 Fig — See Fig 4 for details. (PDF) [file pone.0279225.s009.pdf]

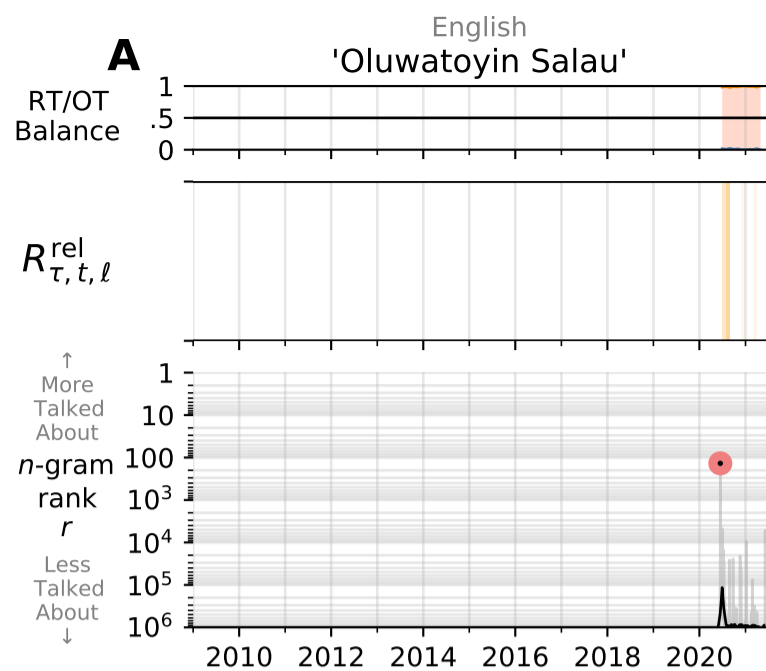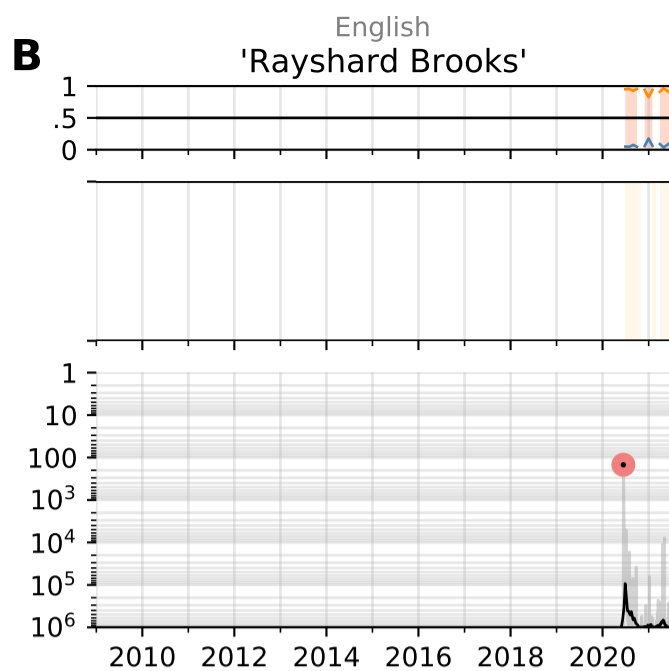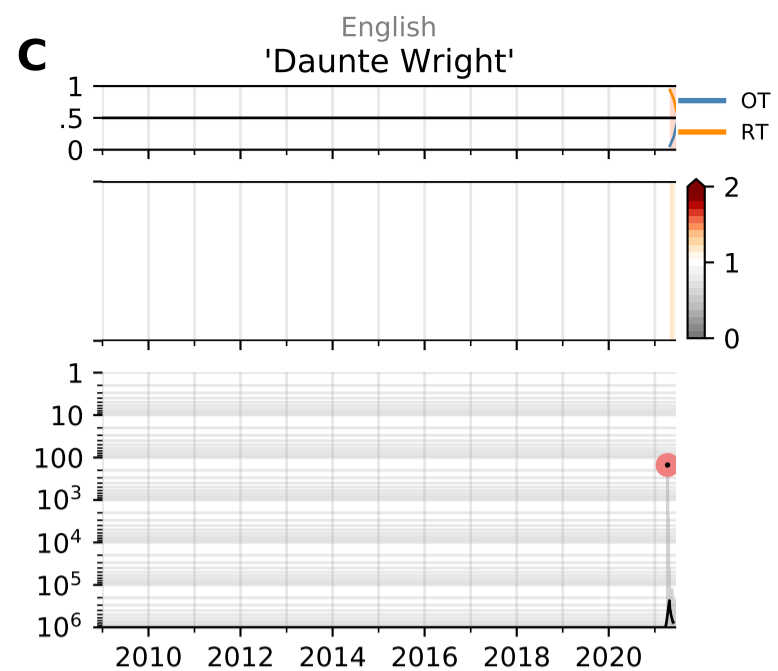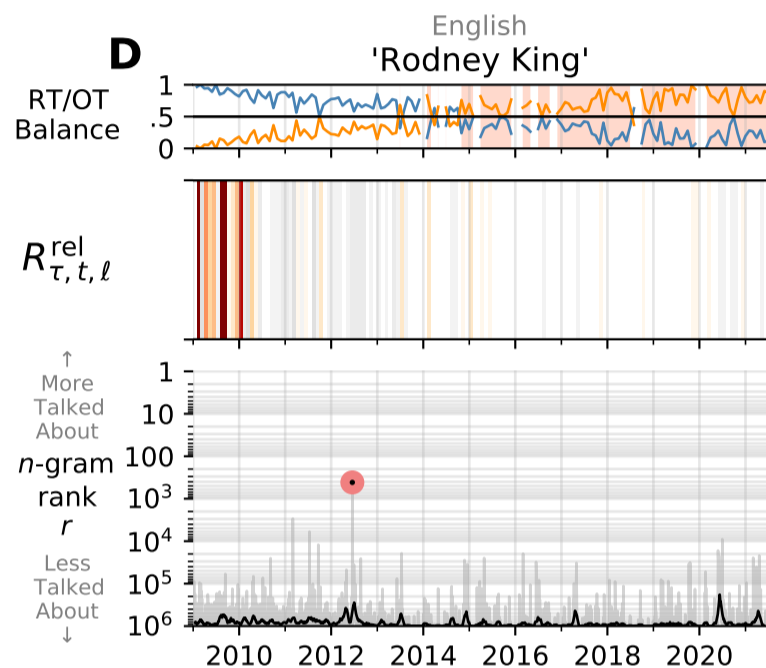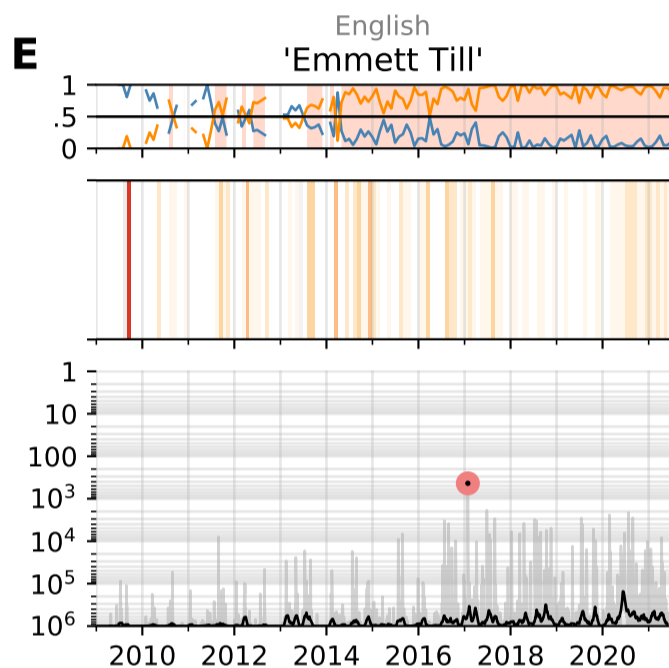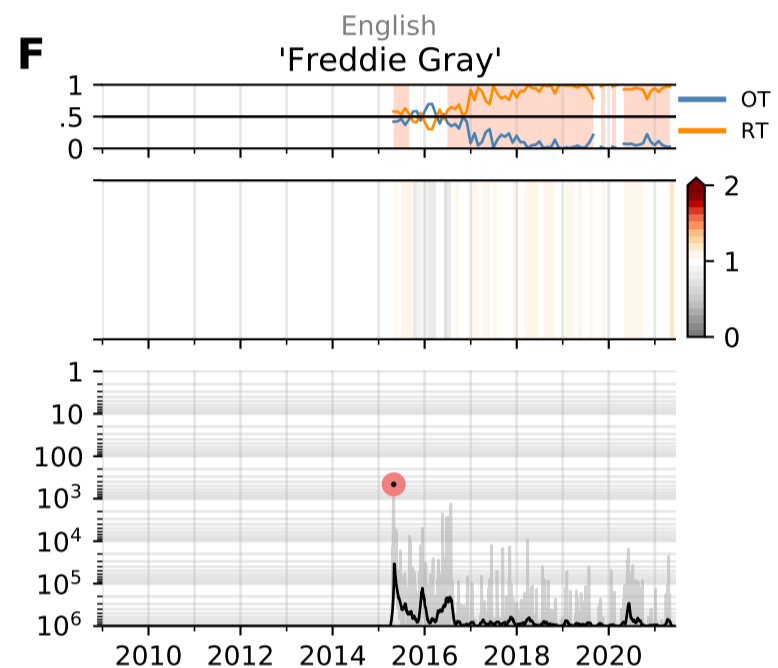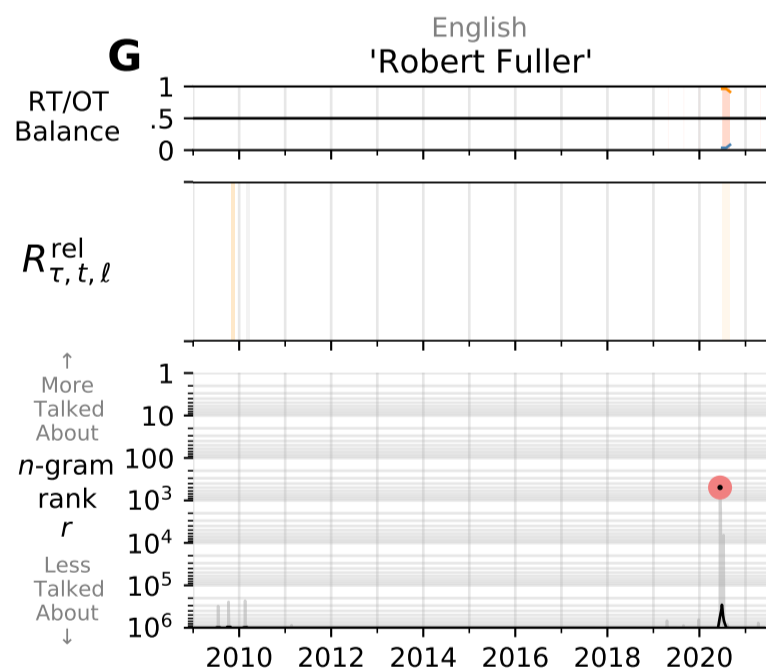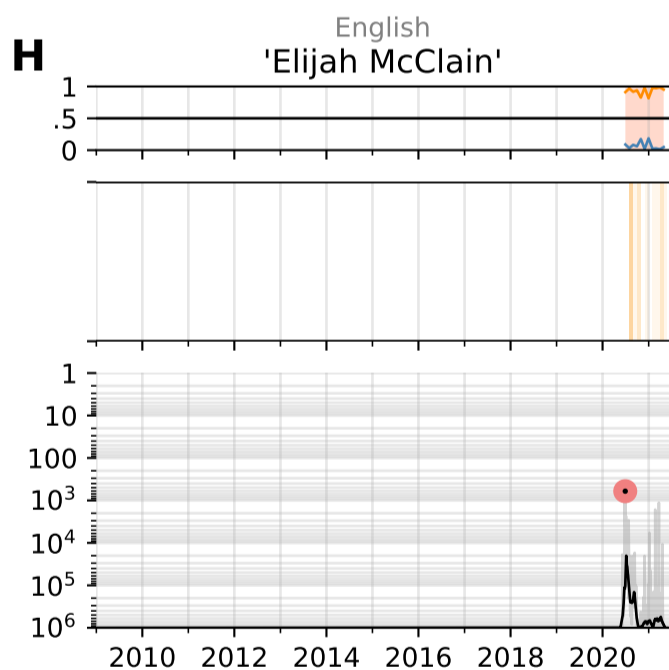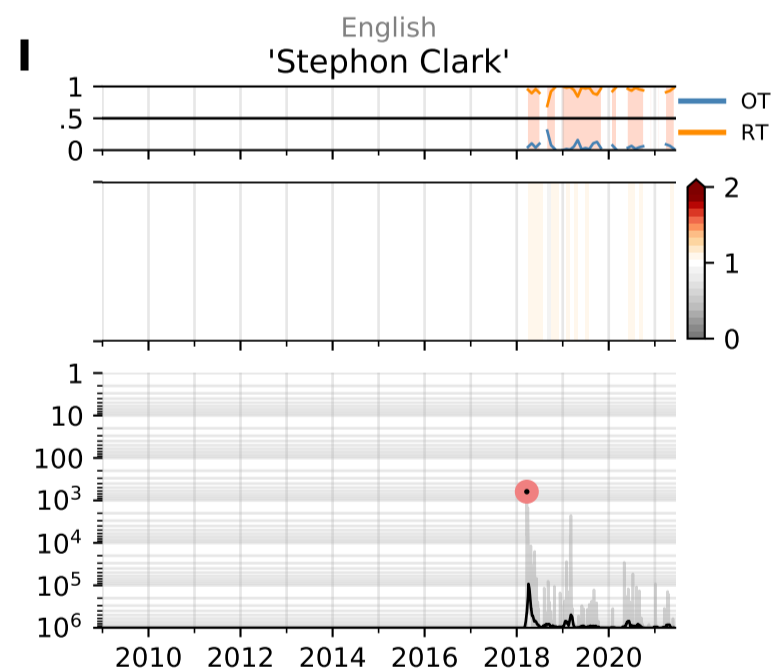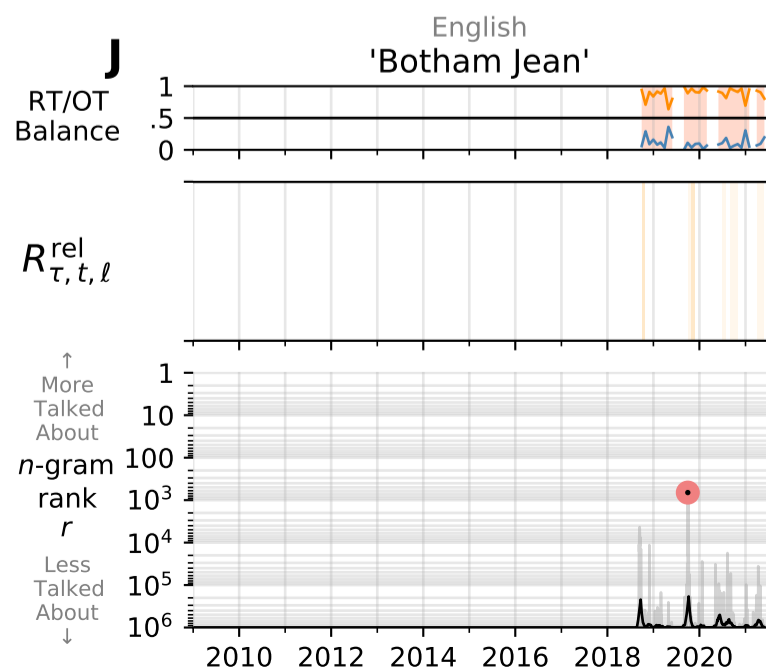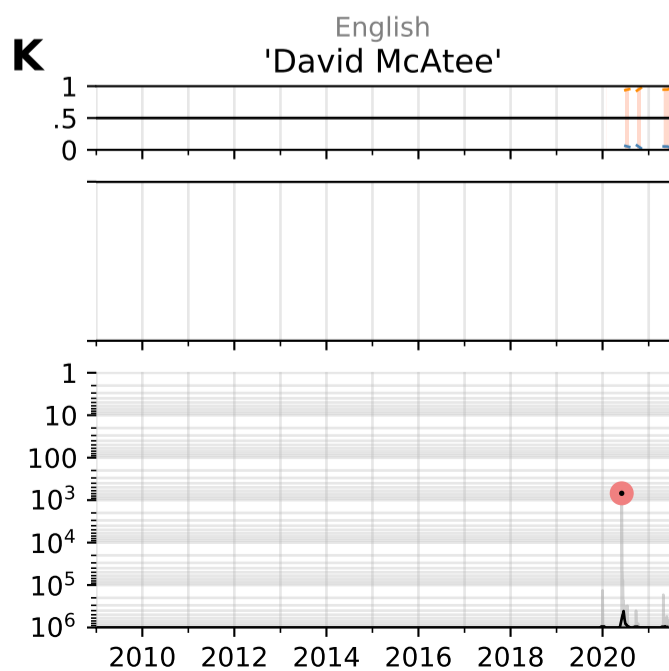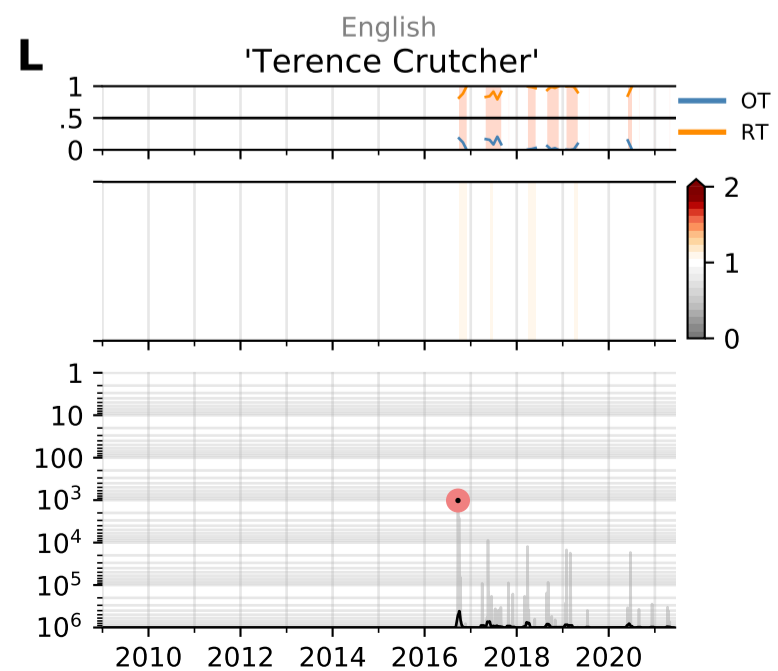

Supplement: S10 Fig — We see patterns similar to those observed in Fig 5, such as high relative social amplification (> 1) and dual peaks of attention. (PDF) [file pone.0279225.s011.pdf]

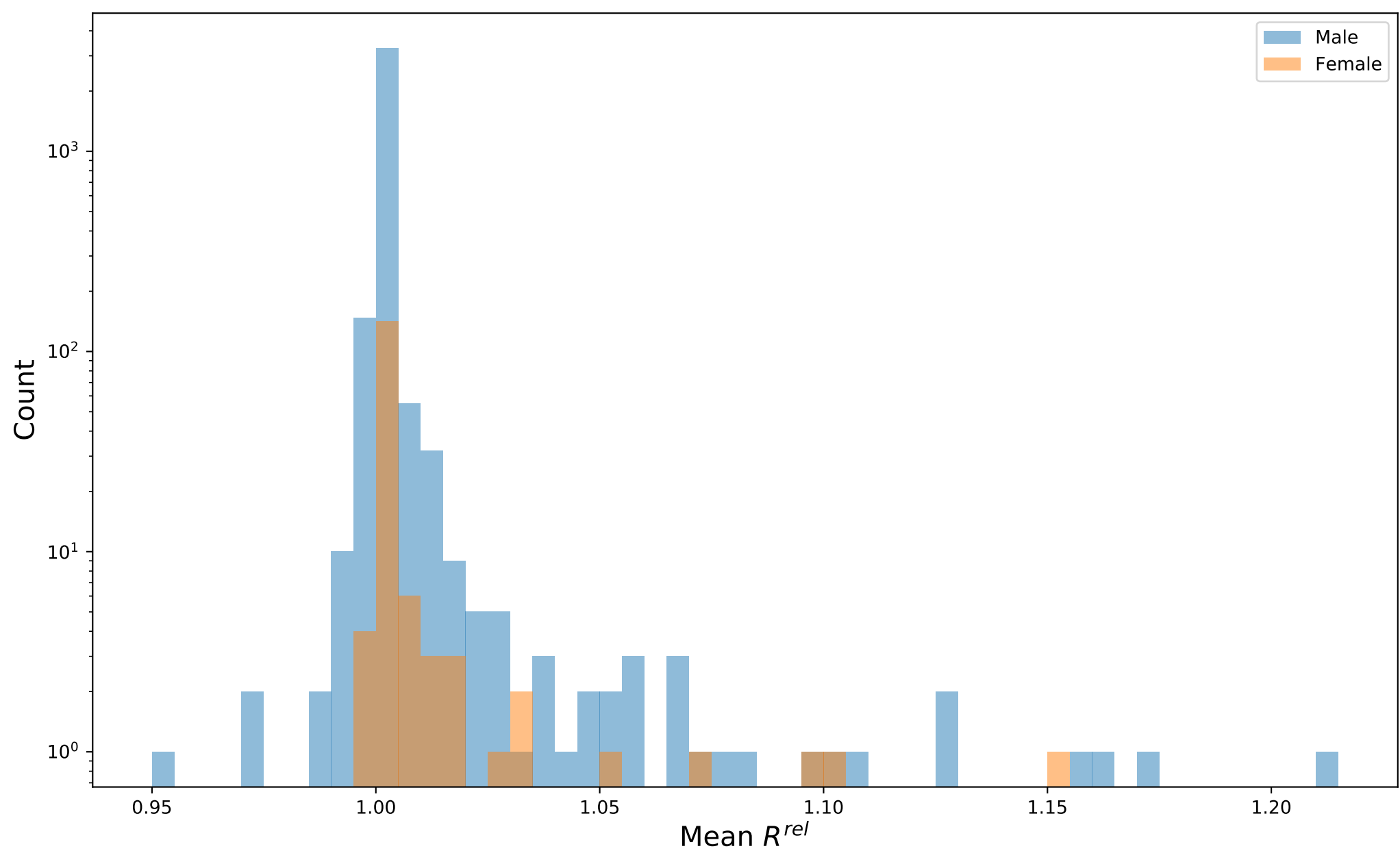

Supplement: S11 Fig — Although there are many more men than women in our analysis, their distributions of mean relative social amplification are similar. Many are concentrated around 1 and the distributions skew right, with more values above 1 than below it. (PDF) [file pone.0279225.s012.pdf]

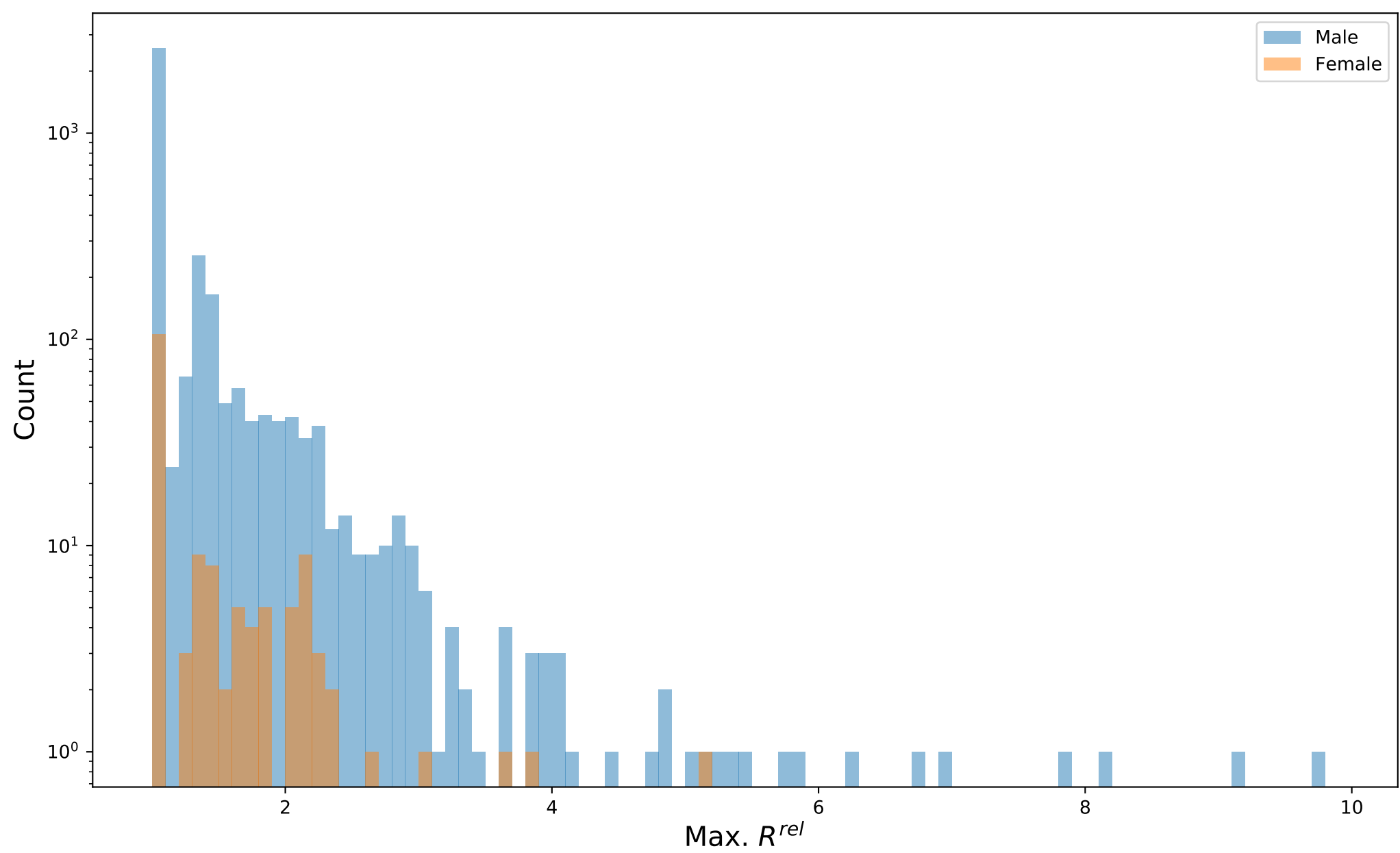

Supplement: S12 Fig — The bin size is 0.1. Although there are many more men than women in our analysis, their distributions of maximum relative social amplification are similar. The distributions skew right. There are some outliers (not shown), all men, with a maximum relative social amplification above 10. (PDF) [file pone.0279225.s013.pdf]
