# Supplementary material for: Say their names: Resurgence in the collective attention toward Black victims of fatal police violence following the death of George Floyd
Source: PLoS One. 2023 Jan 11;18(1):e0279225. doi: 10.1371/journal.pone.0279225 (PMC9833594; doi:10.1371/journal.pone.0279225)
Supplement: S9 Fig — See Fig 4 for details. (PDF) [file pone.0279225.s010.pdf]

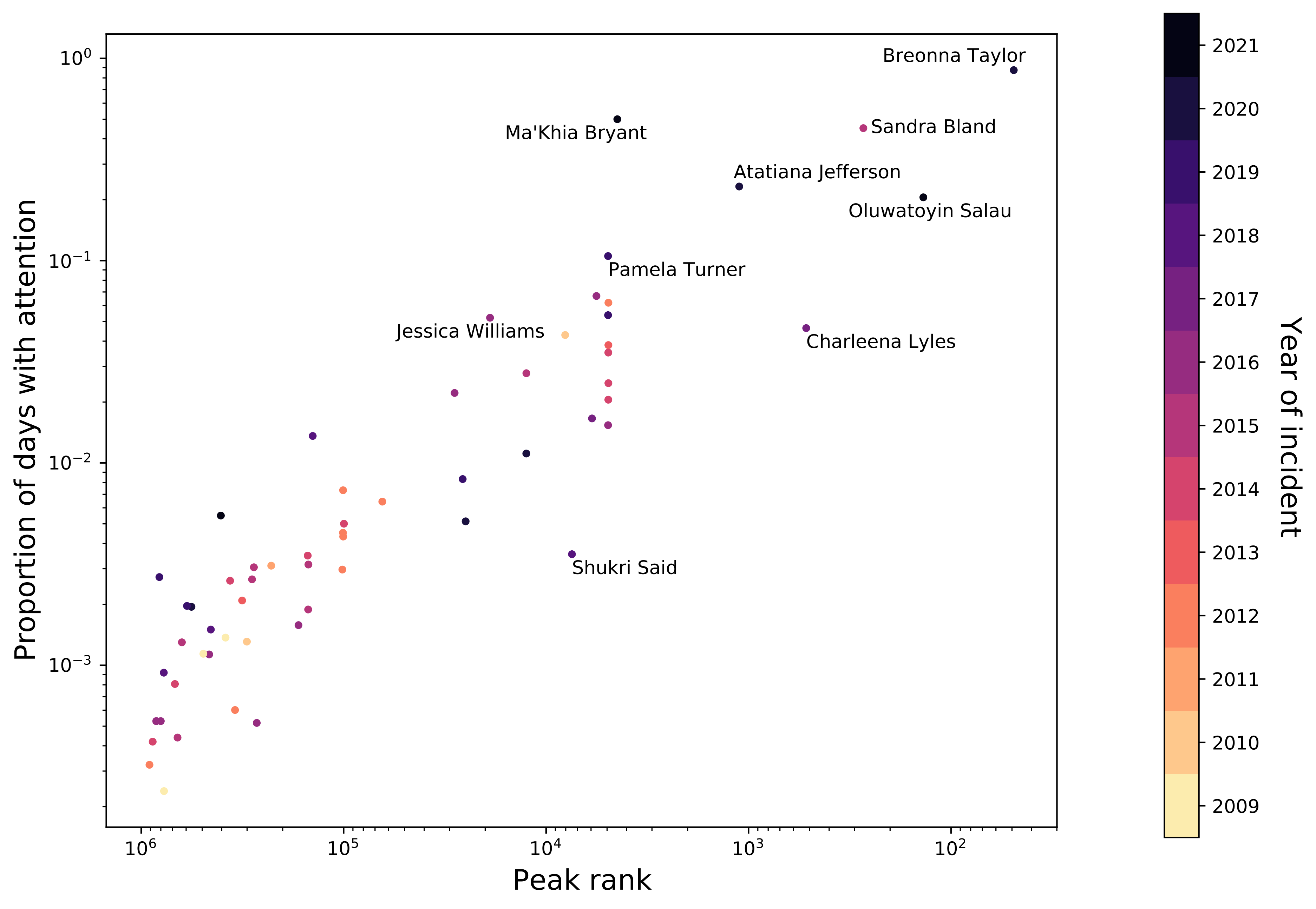

Breonna Taylor
Ma'Khia Bryant
Sandra Bland
Atatiana Jefferson
Oluwatoyin Salau
Pamela Turner
Jessica Williams
Charleena Lyles
Shukri Said
Proportion of days with attention
10^0
10^{-1}
10^{-2}
10^{-3}
10^6
10^5
10^4
10^3
10^2
Peak rank
2021
2020
2019
2018
2017
2016
2015
2014
2013
2012
2011
2010
2009
Year of incident
