# Supplementary material for: Say their names: Resurgence in the collective attention toward Black victims of fatal police violence following the death of George Floyd
Source: PLoS One. 2023 Jan 11;18(1):e0279225. doi: 10.1371/journal.pone.0279225 (PMC9833594; doi:10.1371/journal.pone.0279225)
Supplement: S1 Table — We attribute all mentions of a name to the earliest incident with a victim of that name, indicated by the date in the table. (PDF) [file pone.0279225.s014.pdf]

Name	Date	Name	Date	Name	Date
William Smith	2009-05-17	Reginald Moore	2015-05-03	Charles Baker	2017-06-02
James Hill	2009-07-23	Richard Davis	2015-05-31	Charles Smith	2018-01-07
Jerome Williams	2011-04-18	Albert Davis	2015-07-17	Michael Ward	2018-03-12
Brenda Williams	2011-04-27	John Allen	2015-11-04	Kenneth Ross	2018-04-11
Dominique Smith	2011-07-30	Charles Smith	2016-01-31	Detandel Pickens	2018-06-23
Robert Thompson	2012-09-01	Eric Harris	2016-02-08	Marcus Smith	2018-09-08
James Coleman	2013-04-24	Kisha Michael	2016-02-21	Ronald Singletary	2018-09-08
Antonio Johnson	2013-07-09	Marquintan Sandlin	2016-02-21	Alonzo Smith	2018-10-10
Kenneth Thompson	2013-08-29	Christopher Davis	2016-02-24	Thomas Johnson	2019-03-19
Brandon Smith	2013-10-13	Michael Wilson	2016-05-22	Gregory Edwards	2019-09-17
William Jackson	2013-12-29	Kendrick Brown	2016-08-13	Anthony Smith	2019-12-26
Charles Brown	2014-04-13	Robert Brown	2016-09-07	Malik Williams	2019-12-31
Warren Robinson	2014-07-05	Gerald Hall	2016-12-25	Richard Davis	2020-01-30
Frederick Miller	2014-08-16	Joshua Jones	2017-01-20	Joshua Johnson	2020-04-22
Andre Jones	2014-08-18	Alonzo Ashley	2017-02-11	Michael Harris	2020-08-29
Kevin Davis	2014-12-29	Eddie Davis	2017-03-23	Anthony Jones	2020-10-12
Brandon Jones	2015-03-19	Kenneth Johnson	2017-04-12	Robert Howard	2021-01-06
Eric Harris	2015-04-02	Darius Smith	2017-05-26	James Alexander	2021-04-07

Table S1. *Duplicate names in the Fatal Encounters database.* We attribute all mentions of a name to the earliest incident with a victim of that name, indicated by the date in the table.
