# Supplementary material for: Say their names: Resurgence in the collective attention toward Black victims of fatal police violence following the death of George Floyd
Source: PLoS One. 2023 Jan 11;18(1):e0279225. doi: 10.1371/journal.pone.0279225 (PMC9833594; doi:10.1371/journal.pone.0279225)
Supplement: S2 Table — A person is said to have received measurable attention in the ten days prior to their death if their name was among the top million 2-grams in the ten days prior. This indicates that the name may be shared with another prominent figure, and so they excluded from the analysis. Note that “Walter Scott” was later added manually because the attention given to the victim of police violence greatly exceeded other usages of the name. (PDF) [file pone.0279225.s015.pdf]

Name	Date	Name	Date	Name	Date
Lamar Smith	2009-01-09	James Brown	2013-01-25	Terrance Williams	2016-11-17
Robert Johnson	2009-02-10	John Harris	2013-03-14	George Bush	2016-11-21
Kevin Jackson	2009-04-15	William Morris	2013-04-02	Frank Clark	2016-11-22
Kenneth Williams	2009-05-10	John Williams	2013-11-14	William Boyette	2017-02-07
Michael Williams	2009-06-29	Jason White	2013-11-17	Christopher Carter	2017-02-19
Robert Brown	2009-09-06	Paul Smith	2014-01-14	Don Johnson	2017-03-23
Robert Johnson	2009-09-24	Dustin Brown	2014-01-21	Marcus Williams	2017-04-01
Erik Johnson	2009-10-27	David Robinson	2014-03-10	David Jones	2017-06-08
Maurice Clemmons	2009-12-01	Gary Smith	2014-05-11	Paul Jones	2017-11-09
Michael McIntyre	2009-12-29	James White	2014-05-12	John Doe	2017-11-10
Danny Thomas	2010-02-04	Michael Myers	2014-05-24	Jean Pierre	2017-12-06
Jason Jones	2010-05-01	Samuel Johnson	2014-06-25	Danny Thomas	2018-03-22
Stephen Hill	2010-06-05	Jerry Brown	2014-07-01	Robert White	2018-06-11
Michael White	2010-06-15	Justin Johnson	2014-08-01	Tafahree Maynard	2018-10-22
David Brown	2010-06-20	Anthony Brown	2014-08-23	Tony Smith	2018-11-01
David Smith	2010-09-17	Roshad McIntosh	2014-08-26	Isaiah Thomas	2019-02-02
Robert Thomas	2010-11-08	Christopher Anderson	2014-11-03	Corey Johnson	2019-02-04
Stephen Lee	2010-12-15	James Allen	2015-02-07	Jason Williams	2019-03-14
Eric Williams	2011-01-12	Paul Anderson	2015-04-04	Daniel Warren	2019-05-17
Michael Moore	2011-02-18	Walter Scott	2015-04-04	Ronald Davis	2019-09-15
Larry Brown	2011-02-27	Marcus Wheeler	2015-05-20	Jordan Griffin	2019-09-19
Aaron Williams	2011-03-17	James Brown	2015-08-29	Lamar Alexander	2019-12-05
John White	2011-04-15	Jason Day	2015-10-12	David Irving	2020-02-26
Charles Smith	2011-04-16	Tiara Thomas	2015-11-18	Robert Johnson	2020-05-16
Henry Jones	2011-05-08	Robert Covington	2016-03-03	William Johnson	2020-05-21
William Cooper	2011-06-18	James Brown	2016-03-31	Michael Thomas	2020-06-11
Craig Campbell	2011-07-06	Michael Johnson	2016-06-01	Paul Williams	2020-07-07
Lee Dixon	2012-01-06	John Williams	2016-06-12	David Brooks	2020-07-24
Johnny Wright	2012-01-10	Michael Moore	2016-06-13	Julian Lewis	2020-08-07
Travis Williams	2012-04-19	Andre Johnson	2016-07-09	Brandon Milburn	2020-11-11
Michael Moore	2012-06-23	Alvin Ray	2016-07-25	James Johnson	2021-04-07
Xavier Johnson	2013-01-04	Jeffrey Smith	2016-07-28		

Table S2. *Names excluded because they received measurable attention in the ten days prior to death.* A person is said to have received measurable attention in the ten days prior to their death if their name was among the top million 2-grams in the ten days prior. This indicates that the name may be shared with another prominent figure, and so they excluded from the analysis. Note that "Walter Scott" was later added manually because the attention given to the victim of police violence greatly exceeded other usages of the name.
