# Supplementary material for: Say their names: Resurgence in the collective attention toward Black victims of fatal police violence following the death of George Floyd
Source: PLoS One. 2023 Jan 11;18(1):e0279225. doi: 10.1371/journal.pone.0279225 (PMC9833594; doi:10.1371/journal.pone.0279225)
Supplement: S3 Table — The automatic name disambiguation process (see S2 Table) misses some cases where most uses of the name on Twitter clearly relate to someone other than the victim of police violence. We remove these manually. (PDF) [file pone.0279225.s016.pdf]

Name	Date	Justification
Eric Reid	2009-03-31	American football player
Darren Wilson	2009-04-09	Police officer who killed Michael Brown
Kevin White	2009-11-15	American football player
Eddie Jones	2010-04-28	Australian rugby coach
Michael Smith	2012-01-01	American sports journalist
Bobby Moore	2012-08-12	English soccer player
Justin Turner	2012-11-02	American baseball player
Eddie Jones	2012-12-23	Australian rugby coach
James Anderson	2013-01-27	English cricketer
Jeremy Hill	2013-05-21	American football player
James Anderson	2015-09-25	English cricketer
Thomas Lane	2016-02-22	Police officer involved in case of George Floyd

Table S3. *Manually excluded names for disambiguation purposes.* The automatic name disambiguation process (see Supplementary Table S2) misses some cases where most uses of the name on Twitter clearly relate to someone other than the victim of police violence. We remove these manually.
