# Supplementary material for: Say their names: Resurgence in the collective attention toward Black victims of fatal police violence following the death of George Floyd
Source: PLoS One. 2023 Jan 11;18(1):e0279225. doi: 10.1371/journal.pone.0279225 (PMC9833594; doi:10.1371/journal.pone.0279225)
Supplement: S4 Table — Some names in the Fatal Encounters database are “withheld by police”; therefore, we cannot determine their usage on Twitter. We list the date and location of these cases. (PDF) [file pone.0279225.s017.pdf]

Date	Location	Date	Location	Date	Location
2009-05-27	Chicago, IL	2012-11-22	Detroit, MI	2018-03-05	Kansas City, MO
2011-03-17	Fresno, CA	2013-05-25	Baltimore, MD	2019-01-16	Troy, AL
2011-03-27	Chicago, IL	2013-08-20	Los Angeles, CA	2020-07-23	Detroit, MI
2011-10-08	Baltimore, MD	2016-03-15	Tucson, AZ	2020-11-15	Inglewood, CA
2012-03-31	Chicago, IL	2017-11-15	Jackson, MS		

Table S4. *Cases where the name of the victim was withheld.* Some names in the Fatal Encounters database are "withheld by police"; therefore, we cannot determine their usage on Twitter. We list the date and location of these cases.
