# Supplementary material for: Say their names: Resurgence in the collective attention toward Black victims of fatal police violence following the death of George Floyd
Source: PLoS One. 2023 Jan 11;18(1):e0279225. doi: 10.1371/journal.pone.0279225 (PMC9833594; doi:10.1371/journal.pone.0279225)
Supplement: S5 Table — (PDF) [file pone.0279225.s018.pdf]

Name	Date	Justification
Emmett Till	1955-08-28	Death before timeframe
Rodney King	1991-03-03	Injury before timeframe
Amadou Diallo	1999-02-04	Death before timeframe
Sean Bell	2006-11-25	Death before timeframe
Trayvon Martin	2012-02-26	Non-police-involved death
Jordan Davis	2012-11-23	Non-police-involved death
Natasha McKenna	2015-02-08	Death while in police custody
Walter Scott	2015-05-04	Measurable attention to name prior to death
Kalief Browder	2015-06-06	Suicide related to racism in criminal justice system
Sandra Bland	2015-07-13	Suicide while in police custody
Terrence Crutcher	2016-09-16	Commonly misspelled name
Ahmaud Arbery	2020-02-23	Non-police-involved death
Oluwatoyin Salau	2020-06-06	Non-police-involved death
Robert Fuller	2020-06-10	Ruled suicide under suspicious circumstances
Jacob Blake	2020-08-23	Notable injury

Table S5. *Names that were manually added to the analysis.*
