# Supplementary material for: Say their names: Resurgence in the collective attention toward Black victims of fatal police violence following the death of George Floyd
Source: PLoS One. 2023 Jan 11;18(1):e0279225. doi: 10.1371/journal.pone.0279225 (PMC9833594; doi:10.1371/journal.pone.0279225)
Supplement: S6 Table — We consider four periods of spikes in attention relevant to #BlackLivesMatter: November 24th–December 8th, 2014 (deaths and non-indictments in the cases of Michael Brown, Tamir Rice, and Eric Garner), July 13th–July 26th, 2015 (death of Sandra Bland), July 5th–July 13th, 2016 (deaths of Philando Castile and Alton Sterling), August 12th–August 22nd, 2017 (“Unite the Right” Charlottesville rally), and May 25th–June 6th, 2020 (death of George Floyd). We vary the period before and after the spike across n = 7, 60, and 90 days. The number of names that received increased attention during a spike period is reported, as well as the percentage of those that had not received any measurable attention in the n days prior to the spike. The average change in average relative frequency is calculated for the difference between n days before the spike period and during it, and n days before and after it. Statistical significance is indicated by * for α = 0.05 and ** for α = 0.01. (PDF) [file pone.0279225.s019.pdf]

**Spike Period**	# Names with Increased Attention	% Names w/No Atten. n Days Before	Avg. Diff. in Rel. Freq. Spike - Before	Avg. Diff. in Rel. Freq. After - Before
**7 days**				
Nov. 24–Dec. 8, 2014	87	78.1%	3.73e-08	8.76e-09
Jul. 13–Jul. 26, 2015	46	73.9%	2.39e-09	**1.36e-09****
Jul. 5–Jul. 13, 2016	70	81.4%	**7.94e-09***	4.55e-09
Aug. 12–Aug. 22, 2017	38	89.4%	8.53e-10	4.60e-10
May 25–Jun. 6, 2020	191	92.6%	**5.71e-08****	5.21e-08
**60 days**				
Nov. 24–Dec. 8, 2014	78	56.4%	3.77e-08	2.41e-10
Jul. 13–Jul. 26, 2015	36	55.5%	-3.62e-11	-6.64e-10
Jul. 5–Jul. 13, 2016	66	56.0%	6.63e-09	**1.82e-09****
Aug. 12–Aug. 22, 2017	34	32.3%	-3.64e-09	-4.16e-09
May 25–Jun. 6, 2020	183	77.0%	**5.65e-08****	2.45e-08
**90 days**				
Nov. 24–Dec. 8, 2014	76	47.3%	3.73e-08	-6.85e-10
Jul. 13–Jul. 26, 2015	36	16.6%	-2.39e-09	-3.34e-09
Jul. 5–Jul. 13, 2016	62	48.3%	6.72e-09	1.47e-09
Aug. 12–Aug. 22, 2017	34	27.7%	-2.60e-09	-3.32e-09
May 25–Jun. 6, 2020	184	72.2%	**5.81e-08****	1.89e-08

Table S6. *Robustness of resurgent attention to past victims of police violence following George Floyd's death.* We consider four periods of spikes in attention relevant to #BlackLivesMatter: November 24th–December 8th, 2014 (deaths and non-indictments in the cases of Michael Brown, Tamir Rice, and Eric Garner), July 13th–July 26th, 2015 (death of Sandra Bland), July 5th–July 13th, 2016 (deaths of Philando Castile and Alton Sterling), August 12th–August 22nd, 2017 ("Unite the Right" Charlottesville rally), and May 25th–June 6th, 2020 (death of George Floyd). We vary the period before and after the spike across $n = 7, 60$, and 90 days. The number of names that received increased attention during a spike period is reported, as well as the percentage of those that had not received any measurable attention in the n days prior to the spike. The average change in average relative frequency is calculated for the difference between n days before the spike period and during it, and n days before and after it. Statistical significance is indicated by * for $\alpha = 0.05$ and ** for $\alpha = 0.01$.
