# Supplementary material for: Say their names: Resurgence in the collective attention toward Black victims of fatal police violence following the death of George Floyd
Source: PLoS One. 2023 Jan 11;18(1):e0279225. doi: 10.1371/journal.pone.0279225 (PMC9833594; doi:10.1371/journal.pone.0279225)
Supplement: S7 Table — A name received increased attention if its the mean relative frequency from May 25 to June 7, 2020 was greater than its mean relative frequency from April 25 to May 24, 2020. (PDF) [file pone.0279225.s020.pdf]

Name	Date	Name	Date
Sean Bell	2009-01-01	Joshua Johnson	2013-05-20
Oscar Grant	2009-01-01	Larry Jackson	2013-07-26
Amadou Diallo	2009-01-01	John Allen	2013-08-29
Rodney King	2009-01-01	Brian Nichols	2013-09-03
Emmett Till	2009-01-01	William Brown	2013-09-10
William Smith	2009-01-12	Jonathan Ferrell	2013-09-14
Eric Reid	2009-03-31	Miriam Carey	2013-10-03
Tony Anderson	2009-06-24	William Harvey	2013-10-27
Charles Brown	2009-07-08	Robert Brown	2013-11-25
James Miller	2009-10-21	Jason Lewis	2013-11-27
Kevin White	2009-11-15	Kenneth Herring	2013-12-12
Christopher Wright	2010-02-28	Gregory Hill	2014-01-14
Aiyana Jones	2010-05-16	Jordan Baker	2014-01-16
Kemp Yarborough	2011-03-08	James Norris	2014-02-05
Jerry Moore	2011-06-17	Yvette Smith	2014-02-16
Maurice Hampton	2011-06-30	Victor White	2014-03-03
Kenneth Chamberlain	2011-11-19	Dontre Hamilton	2014-04-30
Malik Williams	2011-12-10	Pearlie Golden	2014-05-06
Wayne Williams	2011-12-22	Eric Harris	2014-06-15
Lawrence Jones	2011-12-23	Lavon King	2014-06-24
Stephon Watts	2012-02-01	Eric Garner	2014-07-17
Ramarley Graham	2012-02-02	John Crawford	2014-08-05
Trayvon Martin	2012-02-26	Michael Brown	2014-08-09
Wendell Allen	2012-03-07	Ezell Ford	2014-08-11
Shereese Francis	2012-03-15	Dante Parker	2014-08-12
Rekia Boyd	2012-03-21	Michelle Cusseaux	2014-08-14
Kendrec McDade	2012-03-24	Kajieme Powell	2014-08-19
James Weldon	2012-04-10	Darrien Hunt	2014-09-10
Alan Blueford	2012-05-06	Laquan McDonald	2014-10-20
Derrick Gaines	2012-06-05	Aura Rosser	2014-11-09
Christopher Brown	2012-06-13	Tanisha Anderson	2014-11-13
Shantel Davis	2012-06-14	Akai Gurley	2014-11-20
Matthew Henderson	2012-07-13	Tamir Rice	2014-11-22
Alesia Thomas	2012-07-22	Rumain Brisbon	2014-12-02
Trevor Taylor	2012-07-31	Jerame Reid	2014-12-30
Bobby Moore	2012-08-12	Natasha McKenna	2015-02-08
Anthony Anderson	2012-09-21	Anthony Hill	2015-03-09
Jordan Davis	2012-11-23	Nicholas Thomas	2015-03-24
Malissa Williams	2012-11-29	Mya Hall	2015-03-30
Shelly Frey	2012-12-06	Phillip White	2015-03-31
James Anderson	2013-01-27	Walter Scott	2015-04-04
George Walker	2013-02-01	Don Smith	2015-04-09
Kayla Moore	2013-02-12	Freddie Gray	2015-04-12
Christopher Taylor	2013-02-21	David Felix	2015-04-25
Kimani Gray	2013-03-09	Alexia Christian	2015-04-30
Kenneth Williams	2013-05-01	Brendon Glenn	2015-05-05
Terrance Franklin	2013-05-10	Kalief Browder	2015-06-06

Table S7. *Names of those who received increased attention during the spike following George Floyd's death.* A name received increased attention if its the mean relative frequency from May 25 to June 7, 2020 was greater than its mean relative frequency from April 25 to May 24, 2020.
