# Supplementary material for: Say their names: Resurgence in the collective attention toward Black victims of fatal police violence following the death of George Floyd
Source: PLoS One. 2023 Jan 11;18(1):e0279225. doi: 10.1371/journal.pone.0279225 (PMC9833594; doi:10.1371/journal.pone.0279225)
Supplement: S8 Table — A name received increased attention if its the mean relative frequency from May 25 to June 7, 2020 was greater than its mean relative frequency from April 25 to May 24, 2020. (PDF) [file pone.0279225.s021.pdf]

Name	Date	Name	Date
Sandra Bland	2015-07-13	John Young	2018-12-01
Darrius Stewart	2015-07-17	Christopher Mitchell	2018-12-09
Samuel DuBose	2015-07-19	Jameek Lowery	2019-01-05
Christian Taylor	2015-08-07	Jacob Harris	2019-01-11
Redel Jones	2015-08-12	Jimmy Atchison	2019-01-22
Jean Charles	2015-08-14	Joshua Williams	2019-02-08
Ricky Ball	2015-10-16	Willie McCoy	2019-02-09
Jamar Clark	2015-11-15	Mario Clark	2019-02-14
Mario Woods	2015-12-02	Osaze Osagie	2019-03-20
Michael Noel	2015-12-21	Isaiah Lewis	2019-04-29
Bettie Jones	2015-12-26	Pamela Turner	2019-05-13
Che Taylor	2016-02-21	Dominique Clayton	2019-05-19
Matthew Tucker	2016-05-04	Miles Hall	2019-06-02
Henry Green	2016-06-06	Ryan Twyman	2019-06-06
Alton Sterling	2016-07-05	JaQuavion Slaton	2019-06-09
Philando Castile	2016-07-06	Brandon Webber	2019-06-12
Micah Johnson	2016-07-07	Eric Logan	2019-06-16
Korryn Gaines	2016-08-01	Isak Aden	2019-07-02
Jamarion Robinson	2016-08-05	Sean Rambert	2019-07-09
Donta Taylor	2016-08-25	Elijah McClain	2019-08-24
Terrence Sterling	2016-09-11	Byron Williams	2019-09-05
Tyre King	2016-09-14	Bennie Branch	2019-09-08
Terence Crutcher	2016-09-16	Atatiana Jefferson	2019-10-12
Terrence Crutcher	2016-09-16	Christopher Whitfield	2019-10-14
Keith Scott	2016-09-20	Dana Fletcher	2019-10-27
Deborah Danner	2016-10-18	David Smith	2019-10-28
Quanice Hayes	2017-02-09	Mark Sheppard	2019-11-15
Cordale Handy	2017-03-15	Ariane McCree	2019-11-23
Jordan Edwards	2017-04-29	Michael Dean	2019-12-02
Terrell Johnson	2017-05-10	Cameron Lamb	2019-12-03
Andrew Kearse	2017-05-11	Jamee Johnson	2019-12-14
Marc Davis	2017-06-02	Darius Tarver	2020-01-21
Charleena Lyles	2017-06-18	William Green	2020-01-27
Corey Mobley	2018-01-23	Manuel Ellis	2020-03-03
Ronell Foster	2018-02-13	Donnie Sanders	2020-03-12
Stephon Clark	2018-03-18	Breonna Taylor	2020-03-13
Shukri Said	2018-04-28	Joshua Ruffin	2020-04-08
Marcus-David Peters	2018-05-14	Desmond Franklin	2020-04-09
Marqueese Alston	2018-06-12	Steven Taylor	2020-04-18
Antwon Rose	2018-06-19	Jonas Joseph	2020-04-28
Daniel Hambrick	2018-07-26	Denzel Taylor	2020-04-29
Diamond Ross	2018-08-18	Said Joquin	2020-05-01
Botham Jean	2018-09-06	Finan Berhe	2020-05-07
Willie Simmons	2018-09-28	Yassin Mohamed	2020-05-09
Patrick Kimmons	2018-09-30	Maurice Gordon	2020-05-23
Jemel Roberson	2018-11-11		
Emantic Bradford	2018-11-22		

Table S8. *CONTINUED: Names of those who received increased attention during the spike following George Floyd's death.* A name received increased attention if its the mean relative frequency from May 25 to June 7, 2020 was greater than its mean relative frequency from April 25 to May 24, 2020.
