# Supplementary material for: Say their names: Resurgence in the collective attention toward Black victims of fatal police violence following the death of George Floyd
Source: PLoS One. 2023 Jan 11;18(1):e0279225. doi: 10.1371/journal.pone.0279225 (PMC9833594; doi:10.1371/journal.pone.0279225)
Supplement: Attachment — Submitted filename: response-to-reviewers.pdf [file pone.0279225.s022.pdf]

Reply to Reviews

Paper: Say Their Names: Collective attention toward Black victims of fatal police violence following George Floyd's death

Academic Editor

Comment

The three reviewers have considerable expertise in the substantive and methodological issues under investigation.

Reviewer 1 recommended acceptance but also offered a couple of suggestions. The first refers to the gender of the victims of police killings. The second refers to the broader significance of the study (an issue also raised by reviewer 2).

Reviewer 2 recommended minor revisions and outlined some areas for improvement. Like reviewer 1 they would like to hear more of your thoughts about the broader significance of this specific case study. Was George Floyd–and the social movement it energised–so unusual in quality and quantity that its singularity means that one cannot really generalize to past, current and future instances of social justice movements and their political context? If so, why? If not, what broader insights can be learnt? Referee 2 also offers some thoughts on the distinction between online and offline activism.

Reviewer 3, who like the other two referees is also exceptionally well placed to assess the paper, recommended minor revisions, and made a few points of a methodological/analytical nature. The first refers to quantifying attention relative to appropriate comparison points. The second relates to why some names may have received more attention (and had different temporal dynamics in terms of attention) than other names. The third is about the readability of Figure 1.

Reply:

We thank the editor for these highlights and respond to each comment from the very helpful reviews in the following pages.

Comment

On the basis of these reviews, I am asking for minor revisions . This is an interesting paper and I look forward to reading the revised version. You do not necessarily need to redraft the manuscript in response to each and every comment, but please do detail in a 'response to review' document your thoughts about the points made by the referees.

Reply:

Thank you. Please see comments on the following pages, including locations where we adjusted the manuscript to reflect the reviewer comments.

We believe that our revised manuscript has been improved through the process of addressing feedback from the reviewers, and look forward to hearing back regarding publication.

Reviewer 1

> **Comment**
>
> This essay could benefit from a bit more gender analysis. I appreciate the authors explaining the origins of Say their Names, derived from #SayHerName, but I wanted to see a bit more about how the number of victims remembered and called forth after George Floyd's murder were men.

Reply:

We appreciate the reviewer's desire for a more thorough analysis of gender as related to the topic, but feel that addressing gender properly is out of scope for the current manuscript. It is definitely worth noting that men's names feature more prominently in the post-Floyd era than women's names, but the reasons for that are impossible for us to address given our dataset. Men are more often killed by police officers (and thus more likely to be named both in our data, and in general), and of course there is misogynoir, general sexism, etc.

We have added a sentence to section 4.2:

Of note, despite some notable exceptions, the most invoked names before and after George Floyd's death were names of men, underscoring the SayHerName call to action and its ongoing importance.

> **Comment**
>
> I would like to understand the significance of the saddest day on Twitter, i.e. what new precedents and considerations might be helpful for the platform to develop or attend to sadness should there be another day that is equally sad or sadder.

Reply:

It is indeed remarkable that the wave of protests associated with George Floyd's murder led to the saddest day in Twitter's history, at least as measured by our instrument. This period was notable for several reasons, including the largest observed volume of amplification of content via retweets. Are there learnings regarding policy available here? Maybe, but we are not in a position to make recommendations given our observational findings.

One mechanism for friction Twitter has put in place recently is to ask individuals who have clicked 'retweet' whether they would like to read the article first prior to sharing. Algorithmic content promotion or demotion is an enormous tangle of trouble we don't have capacity to engage with meaningfully in this manuscript, but is of interest for future work.

Comment

The conclusion could benefit with a more declarative call to action for scholars of Twitter data and some more clarity about the significance of these findings and to whom. It's a great paper and really doesn't need much.

Reply:

We thank the reviewer for their support of this work. While we tried to address some of these points in the preceding 'Discussion' section, we agree with the reviewer that these points can be made more salient in the conclusion. To address this we have added two sentences to section 6:

Moreover, we have shown how the quantitatively extraordinary salience of George Floyd's murder is one moment in a broader movement for racial justice and for the dignity of Black lives.

and

We urge scholars of Twitter and other social media data to further investigate not only those factors that lead some Black people subject to police violence to receive less online attention, but also the strategies employed by members of Black counterpublics to advocate for positive social change.

Comment

There is a typo in the abstract with "ever" repeated.

Reply:

Typo has been fixed thank you.

Reviewer 2

Comment

Overall, this is an interesting study. I think it clearly contributes to our knowledge of Black Lives Matter (BLM) in two primary ways. While other scholars have published data showing spikes in attention around BLM after the George Floyd murder, this piece is exceptional (to my knowledge) in specifically studying sentiment on Twitter around Floyd's death and the ways Floyd's name was connected with 185 other individuals as a result of 'say their names' as a discursive strategy. The authors also provide a smart conceptualization of this as a rhetorical/narrative strategy that connects this murder to others, and in turn, creates a longer history of witnessing of police violence.

Reply:

We thank the reviewer for their support for this work.

Comment

For this reason, I think this piece makes a clear and important empirical contribution to our understanding of this particular movement and moment. I am less clear on its theoretical contribution - or how much we can logically generalize beyond this movement, this strategy, this struggle for racial justice, to understand movement tactics more generally, or even conceptualize this particular movement. In other words, where are the analytical or empirical limits of this case. The paper seems to imply that the rhetorical and narrative tactic of say their names, in being grounded in a particular Black tradition of witnessing, is in fact unique to this context - but I think the paper would be well served to come out and say that then explicitly as part of the case conceptualization. Indeed, there are extensive citations to Richardson's book here, but not for its singular theoretical achievement - namely, that scholars need to abandon any generalized theory of witnessing shorn of race, ethnicity, context, history, and power. Alternatively, if that is not the argument (i.e.: that a Black struggle for racial justice is, in fact, singular), the authors would be well served to step outside of the narrowness of their case here and logically generalize to other movements for either racial justice, or other cases of hashtag activism; theoretically, would rhetorical or narrative strategies like this also further movements for justice in other contexts? If not, why not? Why don't we see similar tactics in other movements? Or, if we do, why are they presumably not as successful? This matters, because as it stands this really is solely a descriptive study of a prominent (if not the prominent) racial justice movement. I think it is an important contribution in its own right - that is not at issue. But the piece would be considerably strengthened by at least thinking about this case as a case - what are its connections to other cases, or limits (in the Richardson view) given its singular history?

Reply:

We appreciate the reviewer encouraging us to reflect more deeply on how the findings of our study affect our understanding of other movements. We have added the following to the Discussion:

> By looking at the totality of Black victims of police violence, rather than any single one, we can learn more about how signifiers are used in racial justice activism. The extent to which that activism is embedded in a longer history of bearing witness to anti-Black violence raises deeper questions about how other instances of hashtag activism are and are not able to be as impactful as #BlackLivesMatter. The use of hashtags as a rhetorical protest technique is not unique to the Black Lives Matter movement, nor is it the only movement to successfully use them to achieve visibility [?]. However, #BlackLivesMatter is much more exceptional in its ability to achieve sustainability. While the persistence of #BlackLivesMatter certainly depends upon a myriad of factors, including formal social movement organizing, our study suggests that it is also particularly rooted in Black Twitter as a cultural extension of the Black public sphere [?, ?, ?]. Taking this historically contextual view—where the success of hashtag activism relies upon its ability to draw upon deep, long-running movements and narratives—challenges the view of hashtag activism as spontaneously "viral." For example, rather than viewing the similarly exceptional impact of the hashtag #MeToo as a singular one-off success, it should be seen as the culmination of several preceding hashtag campaigns challenging sexual violence—including #YesAllWomen and #TheEmptyChair [?]—that was best able to tap into the centuries-long momentum of feminist movements. While signifying names as hashtags in the tradition of the Black press is particularly unique to #BlackLivesMatter, our work points toward the potential for future work on hashtag activism to connect seemingly instantaneous online discussions of marginalization and oppression to their deeper histories.

Comment

Empirically, I think the use of the Fatal Encounters database is novel and very interesting. The empirical results around attention and amplification are pretty extraordinary and clearly empirically document what at least seems to be an overarching aim of the movement (and confirm Richardson's broad historical continuity framework). I think the limitations section here is well done - I had similar questions about the use of the Fatal Encounters database (but also know enough about the lack of standardization in police reporting to see its use as being well-justified.) I agree that data on knowing why these names, and not others, would be important. I also agree that there is an open question beyond the descriptive here about just how effective this is as a tactic - how is it connected to wider policy outcomes or power outcomes? Does it set the agenda across other mediums, or in Democratic Party policy-making and elite rhetoric? I think much more could be done to at least discuss these possibilities (drawing in other work on how social movement activism works in tandem with social media and how there is a diverse range of targets and outcomes).

Reply: We agree with the reviewer that there are a number of directions that future work could take on how hashtag activism affects policy making, framing, agenda setting, and other political outcomes. Given the current length of our paper, our goal to describe the online dynamics around

George Floyd and BLM over the past 12 years, and that there are other papers that more directly engage with how online activism has offline impacts, we have elected to keep our current discussion of those future directions at its current length. We have added a brief citation to Freelon et al. 2018 as a particularly relevant example of research that works to connect the BLM movement with offline impacts.

Comment

I would also drop any notion of online vs. offline activism, which sociologists have long argued against. It also blunts the force of the findings here - movements are always online and offline simultaneously, there are no meaningful distinctions, only strategics, tactics, tools, and goals that work in tandem.

Reply: We agree with the reviewer that there is not a clear dichotomy of online and offline activism, as it acts together in a hybrid media system. We have attempted to convey this stance throughout the paper, particularly in Section 2.2, for example:

> Exactly for that reason, journalists look towards Black Twitter and other online communities to understand emerging events and people's reactions to them [26, 27]. Rather than having to appeal directly to print and news media, activists and others can find their messaging distributed more broadly in the public sphere when it is picked up by journalists via social media [28, 29].

and

> Rather than replacing the offline functions of the Black public sphere [14], networked counterpublics' ability to digitally weave many individual experiences into a larger collective tapestry of oppression [33, 35] makes it possible to bear witness to anti-Black violence in new ways that emphasize the scale of the injustice.

We acknowledge there were some places in the manuscript that it could seem as if we were drawing a clear dichotomy between online and offline activism. In those places, we have altered the phrasing to either emphasize that we are comparing online activism to pre-internet offline activism (i.e. to emphasize the unique affordances provided by social media), or to explicitly clarify that we see these two forms of activism as working in tandem. We choose not to completely drop the distinction, as we believe online and offline activism are complementary but not identical.

Reviewer 3

Comment

This article uses data from Twitter to demonstrate that the murder of George Floyd by police in 2020 led to an unprecedented increase in the levels of public attention to the Black Lives Matter movement, which is illustrated specifically by spikes in mentions of the names of the other Black victims of police violence. This is a strong manuscript that offers detailed and novel descriptive evidence about the dynamics of attention and amplification of content related to the Black Lives Matter movement on Twitter.

While other papers or policy briefs (most notably the work by Deen Freelon) have already offered descriptive evidence about the role that social media played in the spread of the the Black Lives Matter movement, my view is that this article represents a major contribution to the literature in three different ways. First, to my knowledge the longitudinal analysis presented here is based on the longest time period analyzed in any previous study. Second, it develops novel new quantitative metrics of attention and amplification that help track how specific words become salient during particular points in time, and how they are spread via retweets, and which I foresee will be used in future work. Finally, the article relies on excellent data visualizations that elegantly display time trends and which, again, I see as a notable methodological contribution to the literature.

Reply:

We thank Reviewer 3 for their strong support of our work.

Comment

Despite these strengths, the article also has some shortcoming that I believe should be addressed before it can be published.

First, the article could do more to contextualize the time trends that are described, and how attention to Black Lives Matter and the names of Black victims of police violence compares to other topics. I understand that many of the metrics are normalized with respect to the most frequent words, but I think more could be done to more clearly illustrate why this level of attention is unprecedented. In page 10 for example we get a few examples about the attention peaks in comparison to other proper names. But what about other common bigrams, such as those related to news events, TV shows, pop stars, etc.?

Reply:

We have edited and expanded our discussion of how George Floyd's name reached the level of the most basic function 2-grams. We have added a paragraph on 1-gram rankings which we have

studied in great detail in Ref [58], and for which we can provide supporting evidence about which entities rise to such high levels of "fame."

We have also removed the word "unprecedented" at the start of the paragraph in question,

The revised paragraph and the added paragraph on 1-grams are as follows:

> The increase in tweet volume during the spike period resulted in "George Floyd" being the 7th most used 2-gram on Twitter on May 29th, 2020. To help convey just how exceptional this rank is [58], we report that only the functional 2-grams "!!", "of the", "??", "in the", ", and", and "to be" appeared more frequently than "George Floyd", with "to the", "on the", and ". I" rounding out the top ten. Only a few dozen other 2-gram proper names have reached similar levels of attention over approximately five thousand days of Twitter between 2008 and 2022. Others include "Muhammad Ali" who reached a rank of 2 on June 4th, 2016, the day after his death, and "Donald Trump" who reached a rank of 6 on November 9th, 2016 the day following his election as president of the United States. Among those who have reached such stratospheric levels of attention before May 2020, George Floyd is the only one who was unknown to the public earlier the same week.
>
> To give further grounding, in separate analyses of 1-gram distributions [58], function words durably populate the top 1000 1-grams in English. Over the period of 2015 to 2020, only the K-pop band BTS and Donald Trump maintained ranks in the top 300 on a near daily basis. An analysis of country names used on Twitter in the year 2018 showed that 'America' was the most common with a rank of around 1000. In sum, reaching the top 10 1-gram and 1-gram for a day o Twitter is rare, and sudden jumps to such heights signify a world-scale event.

Comment

Second, by the end of the paper I was somewhat missing a more thorough attempt to describe which names received more attention and which names did not. The authors appear to leave this to future work (see pp.19-20), but I wonder if more could be done here to at least anecdotally describe any initial patterns that emerge from the data, so that future researchers can at least have some priors to build on. Similarly, I would have loved to see more descriptive evidence not only on the spikes, but also on how long attention persists and then decays, and whether it decays at a similar pace for all names.

Reply:

We believe the factors listed on page 20 that may determine the amount of attention a name receives serve as a sufficient starting point for future researchers. For instance, an implied hypothesis is that Black police violence victims will receive more attention when there is video footage of events related to their deaths. It is difficult to ascertain any initial patterns that emerge from our data—there are similar trends in attention peak and decay across genders (as shown in figures S6, S8, and S9). Constructing a probabilistic model to predict the attention that a Black victim of police violence will receive based on various factors would be a significant undertaking best left for other researchers. Regarding descriptive evidence on the persistence and decay of attention, figures S5

and S6 offer some insight into the decay of attention after incidents and dates of peak rank. The wide range of individual data points in these figures show that there is substantial variation in the rate of attention decay between victims.

Comment

Finally, I would encourage the authors to revisit Figure 1, in which they describe the data. It was extremely hard to read, particularly the top facet. The y-axis labels could also be improved by adding more informative labels. I would recommend increasing its size. Also, why does it only start on 2020, compared to other graphs that start much earlier?

Reply:

We thank the reviewer for this good suggestion. We have made the figure larger, especially the top facet, for ease of viewing. The top facet's y-axis label was also changed for clarity. The graph starts in 2020 because it is focused on the spikes in tweet volume and sentiment following the death of George Floyd in finer detail. There are other spikes visible (the beginning of coronavirus restrictions, the January 6 Capitol attack) that serve as reference points for the magnitude of the death of George Floyd on Twitter. Details such as the gray bands (when "Black Lives Matter" was among the top 5,000 most common 3-grams) would be less discernable at a larger time scale. The other figures cover the entirety of Storywrangler's records and place the spike in the context of past events relating to Black Lives Matter.
